# Whole-genome profiling of nasopharyngeal carcinoma reveals viral-host co-operation in inflammatory NF-κB activation and immune escape

**DOI:** 10.1038/s41467-021-24348-6

**Published:** 2021-07-07

**Authors:** Jeff P. Bruce, Ka-Fai To, Vivian W. Y. Lui, Grace T. Y. Chung, Yuk-Yu Chan, Chi Man Tsang, Kevin Y. Yip, Brigette B. Y. Ma, John K. S. Woo, Edwin P. Hui, Michael K. F. Mak, Sau-Dan Lee, Chit Chow, Sharmila Velapasamy, Yvonne Y. Y. Or, Pui Kei Siu, Samah El Ghamrasni, Stephenie Prokopec, Man Wu, Johnny S. H. Kwan, Yuchen Liu, Jason Y. K. Chan, C. Andrew van Hasselt, Lawrence S. Young, Christopher W. Dawson, Ian C. Paterson, Lee-Fah Yap, Sai-Wah Tsao, Fei-Fei Liu, Anthony T. C. Chan, Trevor J. Pugh, Kwok-Wai Lo

**Affiliations:** 1 Princess Margaret Cancer Centre, University Health Network, Toronto, ON Canada; 2 Department of Anatomical and cellular Pathology, The Chinese University of Hong Kong, Hong Kong SAR, China; 3 State Key Laboratory of Translational Oncology, The Chinese University of Hong Kong, Hong Kong SAR, China; 4 School of Biomedical Sciences, The Chinese University of Hong Kong, Hong Kong SAR, China; 5 Department of Computer Science and Engineering, The Chinese University of Hong Kong, Hong Kong SAR, China; 6 Sir Y.K. Pao Centre for Cancer, The Chinese University of Hong Kong, Hong Kong SAR, China; 7 Department of Clinical Oncology, The Chinese University of Hong Kong, Hong Kong SAR, China; 8 Department of Otorhinolaryngology, Head and Neck Surgery, Prince of Wales Hospital, The Chinese University of Hong Kong, Hong Kong SAR, China; 9 Warwick Medical School, University of Warwick, Coventry, UK; 10 Department of Oral & Craniofacial Sciences and Oral Cancer Research and Coordinating Centre, University of Malaya, Kuala Lumpur, Malaysia; 11 School of Biomedical Sciences, Li Ka Shing Faculty of Medicine, The University of Hong Kong, Hong Kong SAR, China; 12 Department of Radiation Oncology, University of Toronto, Toronto, ON Canada; 13 Department of Medical Biophysics, University of Toronto, Toronto, ON Canada; 14 Ontario Institute for Cancer Research, Toronto, ON Canada

**Keywords:** Head and neck cancer, Tumour virus infections, Cancer genomics, Targeted therapies, Tumour-suppressor proteins

## Abstract

Interplay between EBV infection and acquired genetic alterations during nasopharyngeal carcinoma (NPC) development remains vague. Here we report a comprehensive genomic analysis of 70 NPCs, combining whole-genome sequencing (WGS) of microdissected tumor cells with EBV oncogene expression to reveal multiple aspects of cellular-viral co-operation in tumorigenesis. Genomic aberrations along with EBV-encoded LMP1 expression underpin constitutive NF-κB activation in 90% of NPCs. A similar spectrum of somatic aberrations and viral gene expression undermine innate immunity in 79% of cases and adaptive immunity in 47% of cases; mechanisms by which NPC may evade immune surveillance despite its pro-inflammatory phenotype. Additionally, genomic changes impairing *TGFBR2* promote oncogenesis and stabilize EBV infection in tumor cells. Fine-mapping of *CDKN2A/CDKN2B* deletion breakpoints reveals homozygous *MTAP* deletions in 32-34% of NPCs that confer marked sensitivity to MAT2A inhibition. Our work concludes that NPC is a homogeneously NF-κB-driven and immune-protected, yet potentially druggable, cancer.

## Abstract (web summary)

The genomic characterisation of nasopharyngeal carcinoma (NPC) remains crucial. Here, the authors perform whole-genome sequencing for 70 NPCs with EBV gene expression, report the somatic alterations and EBV-mediated effects converging on NF-κB activation and immune escape and identify targetable homozygous MTAP deletions.

## Introduction

Nasopharyngeal carcinoma (NPC) is an etiologically complex tumor with a unique combination of epidemiologic, histologic, and viral features. NPC afflicts ~129,000 new patients each year (GLOBOCAN 2018), with the highest incidence occurring in South-East Asia, North Africa, and in the Inuit population in the northern regions of North America^1–3^. NPC is characterized by poorly differentiated malignant epithelial cells residing in a complex microenvironment with heavy lymphocyte infiltration, giving rise to the appearance of a lymphoepithelioma with an apparent inflammatory phenotype. Malignant cells are uniformly positive for the Epstein-Barr virus (EBV), a herpesvirus widespread in the human population. EBV persistent latent infection of B lymphocytes is causally linked to several B-cell malignancies including Burkitt lymphoma, Hodgkin disease, AIDS-related lymphoma, diffuse large B-cell lymphoma, along with a somewhat unexpected association with epithelial tumors such as NPC and a small subset of gastric carcinomas.

Recent functional and genomic studies have implicated NF-κB pathway activation (e.g., *TNFAIP3, MYD88*) and immune evasion (e.g., *PDL1/CD274, PDL2/PDCD1LG2*) in the pathogenesis of EBV-associated lymphomas^4–7^. However, there remains little understanding of how somatic genetic aberrations and viral infection co-operate in the malignant process. In a paradox particular to NPC, it is unclear how viral and somatic factors may interact to establish the tumor’s inflammatory phenotype while rendering the malignant cells apparently obscured from detection by host immunity. Furthermore, the consistent presence of EBV in NPC is puzzling given that the epithelial cells from which NPC derives do not normally sustain a latent virus infection^3,4^. This raises a long-standing question as to whether NPC development involves cellular genetic changes that favor EBV persistence.

As an immediate background to the present study, whole-exome, genome, and targeted DNA-sequencing studies of primary and recurrent NPCs have revealed various somatic alterations that drive constitutive nuclear factor kappa B (NF-κB) signaling activation in up to 40% of cases^8–14^. Our previous work, in this context, isolating the malignant NPC cells from their abundant lymphocytic infiltrate by microdissection, used exome sequencing to identify two driver genes in the NF-κB pathway, *TRAF3* and *CYLD*^8^. Both genes are frequently inactivated by focal homozygous deletions or rearrangements, highlighting the importance of large chromosomal aberrations or structural variants (SVs) in NPC development. The complex nature of genomic changes in NPC underscored the need for whole-genome characterization in conjunction with the evaluation of EBV gene expression, to provide further insights into the interplay of somatic aberrations and viral effects in disease pathogenesis.

In this work, we employed whole-genome sequencing (WGS) to comprehensively delineate the genome landscape of 63 microdissected tumors, 5 patient-derived xenografts, and 2 cell lines of NPC. This included analysis of etiology-associated coding and non-coding mutational signatures, detection of recurrent SVs, and fine mapping of chromosome- and gene-level copy changes across the genome. This approach illuminated multiple routes to constitutive NF-κB activation, as well as immune evasion mechanisms, both involving somatic changes and viral factors. We also uncovered a prevalent mutational signature associated with homologous recombination (HR) repair defects in NPC, revealing a prominent involvement of double-stranded DNA repair defects in NPC pathogenesis. Resulting structural alterations implicate two targets with critical impacts: recurrent *TGFBR2* inactivation that promotes EBV persistence in tumor cells and *MTAP* deletion (adjacent to the *CDKN2A/CDKN2B* loci) with marked sensitivity to MAT2A inhibition potentially applicable to a broad subset of NPC patients. Our findings characterize NPC as universally driven by pro-inflammatory NF-κB signaling counterbalanced by multiple mechanisms of immune protection.

## Results

### Mutational signatures and significantly mutated genes

To establish a comprehensive catalog of genomic aberrations in NPC, we conducted WGS of 63 EBV-positive, non-keratinizing microdissected tumors from Southern Chinese patients (58 primary, 4 recurrent, 1 metastatic; Supplementary data 1), as well as 7 recently established cell lines and PDX models^15^. We achieved genome-wide average sequence coverage of 83× for tumors and 51× for paired normal controls. WGS enabled identification of both coding (*n* = 54,588) and non-coding (*n* = 885,442) simple somatic mutations, which include single nucleotide variants (SNVs), small insertions, and deletions (indels) (Supplementary Data 2, Supplementary figure 1). An orthogonal validation rate of 96% (82/85 events with >10% allelic-fraction, Supplementary data 3) was achieved by target-capture deep sequencing.

**Fig. 1:**
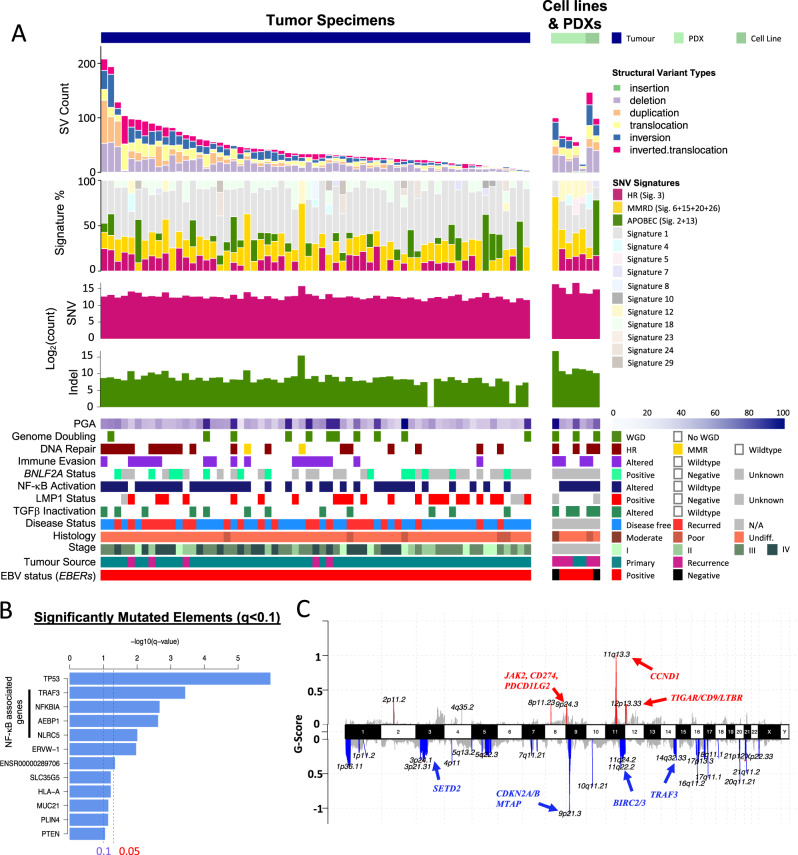
Whole-genome landscape of NPC. **A** Somatic gene alterations and mutational signatures of 70 NPC tumors. For each tumor, the number and types of SNVs and SVs, mutation signatures, whole-genome doubling, and percent genome altered (PGA) were shown in the top panels, as well as somatic changes detected in NF-κB, DNA repair, and TGF-β pathways, expression of EBV-encoded LMP1, *BNLF2a* and *EBERs*, clinical staging and tumor type. **B** Significantly mutated genes and regulatory elements (*q* < 0.1) identified in NPC. The altered genes in NF-κB pathways are indicated. **C** Copy number alterations in NPC. GISTIC copy number variations analysis showing recurrent amplification and deletion of multiple chromosomal regions.

To provide clues regarding NPC etiology, we examined the single base changes observed which confirmed that the predominant type of genome-wide substitution is C>T transitions at NpCpG sites. We also observed recurrent evidence of Signature 3 (present in 64.3% (45/70) cases), which was not reported by previous exome-sequencing studies^8–10^. Mutational Signature 3 is associated with defects in DNA double-strand break repair by HR (Fig. 1A). In addition to HR defects (Signature 3), we found contributions from deamination of 5-methyl-cytosine (Signature 1, 98.6% cases), defective DNA mismatch repair (Signatures 6, 15, 20, and 26, 98.6% cases) and APOBEC/AID (Signatures 2 and 13, 44.3%). Together, these mutational signatures provide insight into NPC development that may underlie the acquisition of specific driver mutations^8–10^. In a recent meta-analysis study of the published exome-sequencing data sets in 402 NPC patients, all four mutational signature classes we identified were confirmed^16^.

To nominate coding and non-coding mutations that drive NPC, we employed ActiveDriverWGS. This identified 11 significantly mutated coding genes and one regulatory region that largely converge on NF-κB signaling (Fig. 1B). Consistent with previous studies, *TP53* was the most significantly mutated gene (*n* = 10; *q* = 1.1 × 10^−6^), followed by *TRAF3*, *NFKBIA*, *AEBP1*, and *NLRC5*; all of which have been reported to regulate NF-κB signaling^4,8–14,17^. In addition, significant somatic aberrations detected in *HLA-A* and *NLRC5* suggest impairment of antigen presentation while *PTEN* mutations may activate the PI3K pathway^8–10^. We also found four coding genes, which were significantly mutated, namely *PLIN4, MUC21, SLC35G5,* and *ERVW-1*. Further studies would be required to define the function of these mutations in NPC.

Among all the SNVs and indels detected, 94% were located in non-coding regions. These include 1480 mutations in putative promoters (The Eukaryotic Promoter Database), 5997 in predicted Enhancers (ENCODE), and 4699 in TF-binding sites (ENCODE). The sole significantly mutated non-coding regulatory region in our cohort, ENSR00000289706, is a CTCF binding site ~16 kb downstream from *CYS1* and ~22 kb upstream of *RRM2* (Fig. 1B). Notably, a single recurrent mutation (chr2:10097565 C > T) was identified in four tumors from three patients (Supplementary figure 2). Despite reports from several genome sequencing studies conducted thus far, this represents the first recurrent mutation identified in the non-coding region of NPC genome^8–14^. Furthermore, this mutation results in a gain of a putative NFKB1/p50 binding sequence upstream of *RRM2* which has been reported to be associated with NF-κB-signaling activation^18^. Of note, mutations in this region were also reported in the International Cancer Genome Consortium (ICGC) pan-cancer data set, including three occurrences of this specific point mutation^19^. Future studies would be required to confirm the predicted functional effect of this and other, less-frequent non-coding mutations.

### Recurrent copy number and structural alterations

**Fig. 2:**
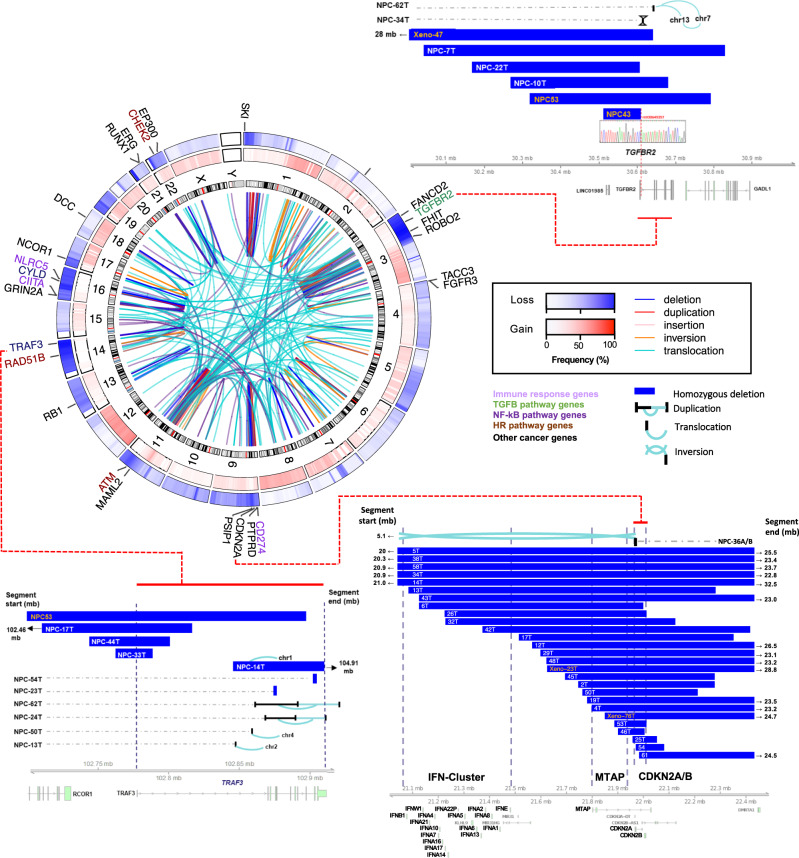
Recurrent structural and chromosomal alterations in NPC. Circos plot showing recurrent SVs and common CNVs in NPC. Frequencies of copy number losses and gains are shown in the outer and inner circles respectively. Selected cancer genes involved in the recurrent inter- and intra-chromosomal SVs (i.e., genes with ≥3 times altered by SVs) are indicated. Homozygous deletions and structural alteration breakpoints identified in *CDKN2A/B* loci, *MTAP*, and cluster of type I IFN genes at 9p13.3, *TGFBR2* at 3p24.1, and *TRAF3* at 14q32.3 are shown. The DNA sequences spanning the breakpoints of deletion of *TGFBR2* identified in NPC43 and Xeno-47 were confirmed by Sanger DNA sequencing.

We identified somatic copy number alterations (SCNAs) using Varscan2 and Sequenza, followed by significance analysis using GISTIC (v2.0.23) (Fig. 1C, supplementary data 4). We detected polyploidy in 15.9% of patient tumors (10/63) and the median percent genome altered (PGA) was 30% (range 9–92%) (Fig. 1C). GISTIC analysis revealed frequent arm-level chromosomal losses and amplification, including frequent gains in chr.1q, 3q, 7q, 8q, 12p, and 12q, and frequent losses in chr. 3p, 9p, 11q, 14q, and 16q (Supplementary figure 3). This analysis defined homozygous deletion of 9p21.3 (*CDKN2A/CDKN2B*) as the most frequently altered chromosome region, impacting 37.1% of cases (Fig. 2)^1,2^. Through the fine mapping of deletion breakpoints within 9p21.3, we noted frequent homozygous deletions of a potential druggable target, *MTAP* (34%), and distally, loss of type I interferon genes (e.g., *IFNA1, IFNA2, IFNA8, IFNE;* 16%) that may be associated with loss of antiviral response in early NPC^2,3^ (Fig. 2). In addition, significantly focal amplified regions harboring potential drivers were found on chr. 11q13 (*CCND1*), 9p24.3 (*JAK2, CD274, PDCD1LG2*) and 12p13.3 (*TIGAR/CD9/LTBR* Fig. 1C). Together, these recurrent SCNAs are consistent with the previous genome-wide studies and accurately defined NPC-associated genes including *CD274* and the type I interferon genes^2,8,20^. Importantly, using microdissected tumor samples and WGS, we detected the highest frequencies of homozygous deletions of the critical tumor suppressor genes *CDKN2A*, *TGFBR2*, *TRAF3,* and potential synthetic lethal target *MTAP* amongst NPC genomic reports thus far^8–14^.

Chromosomal rearrangements were detected using a combination of three SV calling algorithms (Manta (v1.2.2), DELLY (v0.7.7), NovoBreak (v1.1.3)) followed by filtration and annotation using MAVIS (v1.8.4) (Supplementary data 5). Using this approach, we identified a total of 3486 high-confidence SVs, including translocations, inversions, inverted translocations, insertions, deletions, and duplications (Fig. 1A). On average, NPCs harbored 50 SVs per tumor (ranging from 5 to 208), consistent with previously reported rates in head and neck squamous cell carcinoma, lung and colorectal adenocarcinomas (Fig. 1A, suppl. Figure S1). Mapping of recurrent SVs with common chromosomal aberrations revealed substantial overlap with recurrent CNAs including multiple regions on chromosome 3p, 9p, 11q, 14q, and 16q, harboring known tumor suppressors and cancer genes in NPC (Fig. 2). While the heterozygous loss of chr 3p is a known feature of NPC (64/70, 91.4% in our cohort), we also discovered recurrent homozygous deletions and SVs of *TGFBR2* at 3p24.1 in 11.4% of our tumors (Fig. 2). In addition, recurrent breakpoints of SVs targeting cancer genes commonly occurred in frequently deleted regions, e.g., *CDKN2A* on 9p21.3, *MAML2* and *ATM* on 11q21-22, *RAD51B,* and *TRAF3* on 14q 24-32.3, *CYLD* and *NLRC5* on 16q12.1-13 (Fig. 2). Taken together, we conclude that combinations of homozygous deletions and structural alterations are common mechanisms for biallelic inactivation of tumor suppressors in NPC, highlighting the need for comprehensive genome-wide profiling for this cancer type, in particular. Consistent with previous reports, recurrent oncogenic *TACC3-FGFR3* fusions were also detected in our NPC cohort (2/70, 2.9%)^2^.

### EBV and somatic changes co-operate to sustain NF-κB activation

**Fig. 3:**
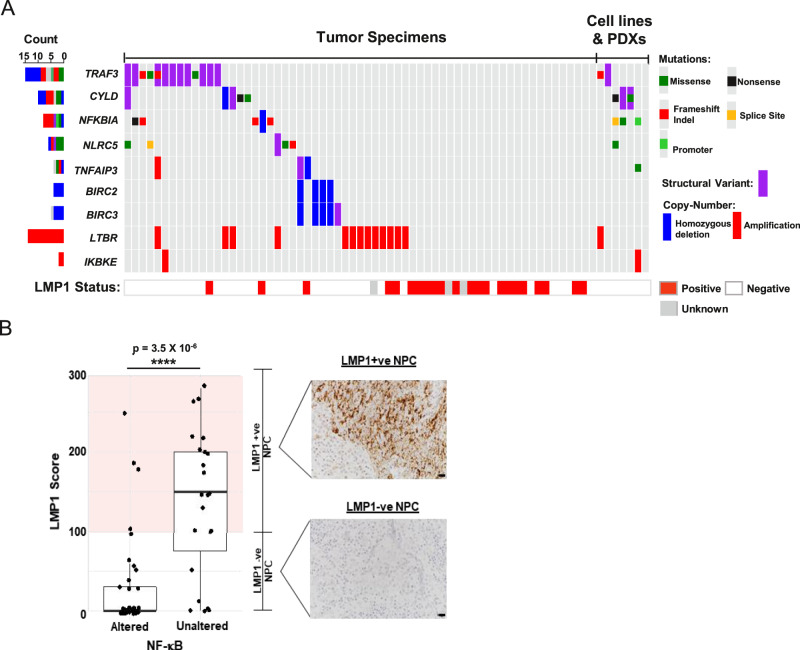
Aberrant NF-κB pathway activated by somatic gene alterations and EBV latent gene. **A** Somatic aberrations activating NF-κB and LMP expression in 70 NPC samples. NPC tumors with LMP1 overexpression are also indicated. **B** LMP1 overexpression and somatic aberrations activating NF-κB pathway are mutually exclusive at the whole-genome level in NPC (two-sided Fisher’s exact test *p* = 3.5 × 10^−6^, ****P < 0.0001). Boxplot is defined as follows: center upper whisker = min(max(x), Q_3 + 1.5 * IQR), lower whisker = max(min(x), Q_1 – 1.5 * IQR); where IQR = Q_3−Q_1, the bounds of the box. LMP1 expression was examined in NPC tumor specimens (*n* = 65) by immunohistochemistry staining. IHC staining and scoring were performed twice in the tumors and similar results were observed. Representative images of NPC cases with high (+ve) or absence/low (−ve) LMP1 expression are shown. Scale bar: 10 μm. Source data are provided as a Source Data file.

By integrating all SNV, SV, and CNV data, we have established a comprehensive catalog of genomic changes implicating multiple oncogenic pathways in NPC. The most prevalently altered pathway encodes NF-κB signaling (44/70; 62.9%) (Figs. 1A and 3A). In addition to our previously reported somatic changes of NF-κB-negative regulators [*TRAF3* (21.4%), *CYLD* (11.4%), *NFKBIA* (10%), *NLRC5* (8.6%), and *TNFAIP3* (5.7%)], we have identified additional alterations impacting this pathway including amplifications of *LTBR* (20%) and recurrent homozygous deletions and rearrangements of *BIRC2* (5.7%) and *BIRC3* (8.6%). In particular, *LTBR* amplification that went unreported in our previous exome study is a demonstrated driver for NF-κB activation in NPC and multiple myeloma^11,20–22^.

The EBV oncoprotein, LMP1, has been demonstrated to drive NF-κB pathway activation in NPC, via tumor necrosis factor receptor/TRAF3 interactions^3^. In this cohort, high LMP1 expression was found in 32.8% (23/70) of tumors (Fig. 3A, B). Previously, we reported the discovery of mutual exclusivity between somatic and LMP1-driven NF-κB-activating events in NPC which we have confirmed herein (Fig. 3B; two-sided Fisher’s exact test *p* = 3.5 × 10^−6^). With additional genome variations, our current WGS study further strengthened this finding, as co-selected NF-κB-activating mechanisms in 90% (63/70) of this tumor cohort with a high degree of mutual exclusivity (two-sided Fisher’s exact test *p* = 7.4 × 10^−7^). Our findings solidly define NPC as a ubiquitously NF-κB-driven malignancy. In addition, LMP1 is also known to activate PI3K/AKT and MAPK signaling pathways^3^. Although rare somatic alterations of PI3K pathways were observed in NPC cases with LMP1 overexpression, similar viral-somatic mutual exclusivity was observed between altered PI3K signaling pathway and LMP1 expression (Supplementary figure 4A, B).

### Impaired immune machinery by EBV and somatic changes

**Fig. 4:**
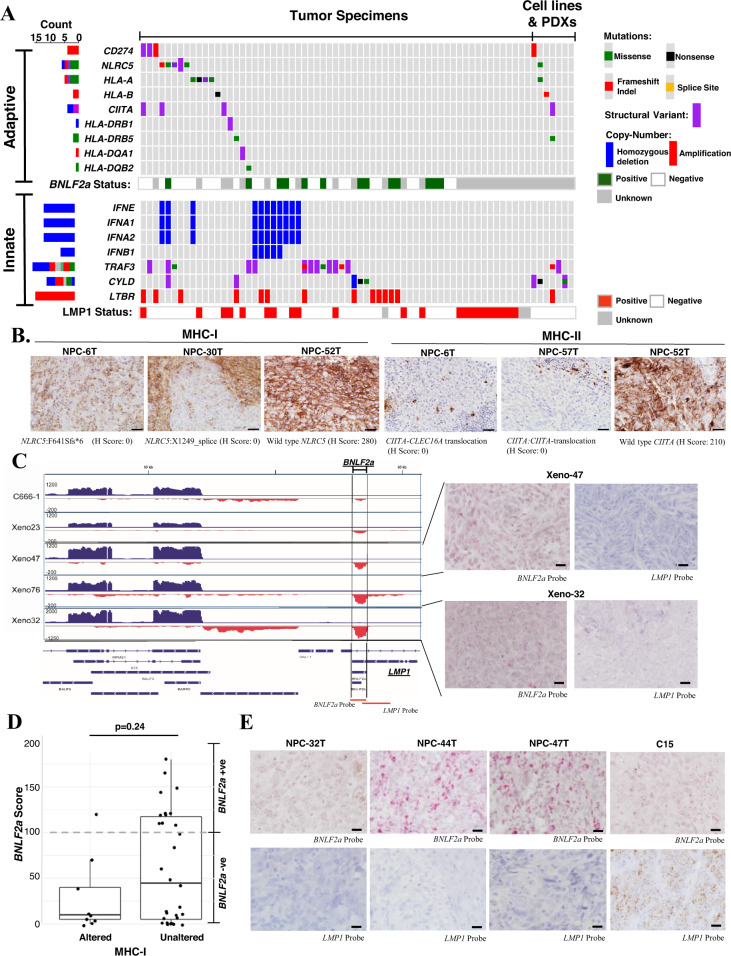
Impaired innate and adaptive immunity by somatic gene alterations and EBV latent gene expression in NPC. **A** Somatic aberrations and EBV-encoded LMP1 and *BNLF2a* expression attenuate adaptive and innate immunities in NPC are shown. **B** By immunohistochemistry analysis, loss of MHC-class I and MHC-class II expression are demonstrated in representative NPC cases with somatic alterations of *NLRC5* (*n* = 3; NPC-6T, NPC-30T, NPC-52T) and *CIITA* (*n* = 3; NPC-6T, NPC-52T, NPC-57T), respectively. The experiments were performed twice and similar results were found. Representative images of MHC-class I and MHC-class II expression in the NPC cases with wild-type or somatic alterations of *NLRC5* and *CIITA* genes are illustrated. Scale bar: 20 μm. **C** RNA-sequencing revealed the expression of *BNLF2a* in NPC PDXs. The partial EBV transcription profiles illustrating *BNLF2a* expression in NPC cell line (C666-1) and PDXs (Xeno-23, Xeno-47, Xeno-76, and Xeno-32) were shown. The RISH probe targeted regions (*BNLF2a, LMP1*) in EBV genome are indicated. By RISH analysis, abundant expression of *BNLF2a* transcripts was found in the majority of tumor cells in two NPC PDXs, Xeno-32 and Xeno-47. *BNLF2a* probe was used to illustrate the signal of *BNLF2a* transcripts in the tumor cells with no *LMP1* probe signal. Each tumor was subjected to RISH analysis twice and similar results were found. Representative images of RISH results of *BNLF2a* and *LMP1* in NPCs are illustrated. Scale bar: 10 μm. **D** Enrichment of *BNLF2A-*expressing cases in NPCs without somatic alterations in MHC-class I pathway (two-sided Wilcoxon signed-rank *p* = 0.24). Boxplot is defined as follows: center upper whisker = min(max(x), Q_3+1.5*IQR), lower whisker = max(min(x), Q_1−1.5*IQR); where IQR = Q_3−Q_1, the bounds of the box. *BNLF2A* expression was determined in NPC specimens (*n* = 39) by RISH. **E** Representative NPC tumors (NPC-32T, NPC-44T, NPC-47T) with high *BNLF2a* expression are shown. An LMP1-expressing xenograft C15 was included for RISH analysis using *BNLF2a* and *LMP1* probes. Each tumor was subjected to RISH analysis twice and similar results were found. Scale bar: 10 μm. Source data are provided as a Source Data file.

With inflammatory NF-κB signaling, persistent expression of various viral RNAs (*EBERs*) and immune antigens (e.g., EBNA1, LMP1, LMP2) in nearly all NPC, immune evasion is believed to be critical for NPC pathogenesis. The abundantly expressed *EBER* transcripts are known to activate IRF3 signaling, which induces type I interferons to elicit innate immunity against EBV-infected NPC cells^23–25^. Indeed, using WGS we uncovered genomic losses of multiple types I interferon genes, including *INFA1, IFNA2, IFNA8,* and *IFNE* by a homozygous deletion in 15.7% of NPC (Fig. 2). Importantly, the inactivation of *TRAF3* or *CYLD* by somatic alterations also attenuates IRF3 signaling. Furthermore, *LTBR* amplification and LMP1 overexpression inactivate TRAF3 activity, thereby inhibiting the innate immune responses for EBV infection through impairment of IRF3 signaling and type I interferon production^2,3,23^. In summary, to avoid the EBV-induced innate immune response by attenuating IRF3 signaling, either acquirement of somatic alterations or overexpression of LMP1 were demonstrated in a total of 55/70 (78.6%) clinical specimens (Fig. 4A).

In terms of adaptive immunity, our data revealed SVs and aberrations of immune checkpoint machinery (*PD-L1/CD274;* Supplementary figure 5*)*, and antigen presentation molecules including MHC class I (*HLA-A*, *HLA-B*), class II genes (*HLA-DQA1*, *HLA-DQB2*, *HLA-DRB1*, *HLA-DRB5*), and their major transcriptional activators (*NLRC5* and *CIITA*) in 22/70 (31.4%) of NPCs (Fig. 4A). Such alterations could impair antigen presentation by tumor cells allowing them to escape from immune surveillance. In fact, loss-of-function (LOF) alterations in *NLRC5* and *CIITA* were consistent with the loss of MHC class I and class II expression in NPC tumor cells (Fig. 4B and Supplementary figure 6). Notably, inactivating mutations in MHC class I genes (*NLRC5, HLA-A, HLA-B*) were significantly associated with the presence of APOBEC-related mutational signature (two-sided Fisher’s exact test *p* = 0.002), but not the DNA mismatch repair defect mutational signature. It is likely that the NPCs with high APOBEC3 activity resulting in the presence of the APOBEC mutational signature also acquire inactivating alterations in MHC class I molecules to enable escape from CD8+ cytotoxic T-cell response. Indeed, a significant relationship between mutational burden and alterations in adaptive immunity-related genes including *CD274, NLRC5, HLA-A, HLA-B, CIITA, HLA-DRB1, HLA-DRB5, HLA-DQB2* (two-sided Wilcoxon signed-rank test *p* = 0.00028). These findings suggested that viral factors and somatic events both contribute to defective DNA mismatch repair and APOBEC3 mutational signatures resulting in high TMB, which subsequently favors the selection and clonal expansion of EBV-infected NPC cells bearing somatic alterations in adaptive immunity-related genes.

In addition to somatic events, we also explored potential viral-associated mechanisms for evading host immune responses. Expression of *BNLF2a*, an EBV lytic gene inhibiting the cellular transporter associated with antigen processing (TAP) protein has been shown to be expressed in gastric cancers latently infected with EBV^26,27^. From RNA-sequencing data sets of EBV-associated PDXs, we also observed *BNLF2a* transcription in these NPC models (Fig. 4C, D and Supplementary figure 7). *BNLF2a* expression was confirmed in the majority of latently infected EBV-positive tumor cells in two NPC PDXs using RNA in situ hybridization (RISH) of a *BNLF2a*-specific probe. Because of the overlap of *BNLF2a* and exon 3 of *LMP1* genes, the cases with positive RISH signals for LMP1-specific probe deemed “undetermined”. Nevertheless, high levels of *BNLF2a* transcript alone were detected in at least 13 NPC tumors available for RISH (Fig. 4D).

Notably, a total of 47.1% (33/70) of NPC tumors had either *BNLF2a* expression or somatic aberrations in antigen presentation and immune checkpoint genes. A trend for mutual exclusivity between high tumor expression of *BNLF2a* and somatic alterations for immune evasion was observed (two-sided Fisher’s exact test *p* = 0.24) (Fig. 4D). These complementary viral and somatic events impairing antigen presentation and immune checkpoints may contribute to the clonal selection for the immune escape of EBV-infected cells during NPC development. This observation of impaired innate and adaptive immunity supports the notion that NPC tumors are protected by both viral and somatic mechanisms of immune evasion in patients, representing the second viral-somatic co-selected mechanisms (after NF-κB activation) for NPC tumorigenesis.

### *TGFBR2* loss is a driver event in NPC

**Fig. 5:**
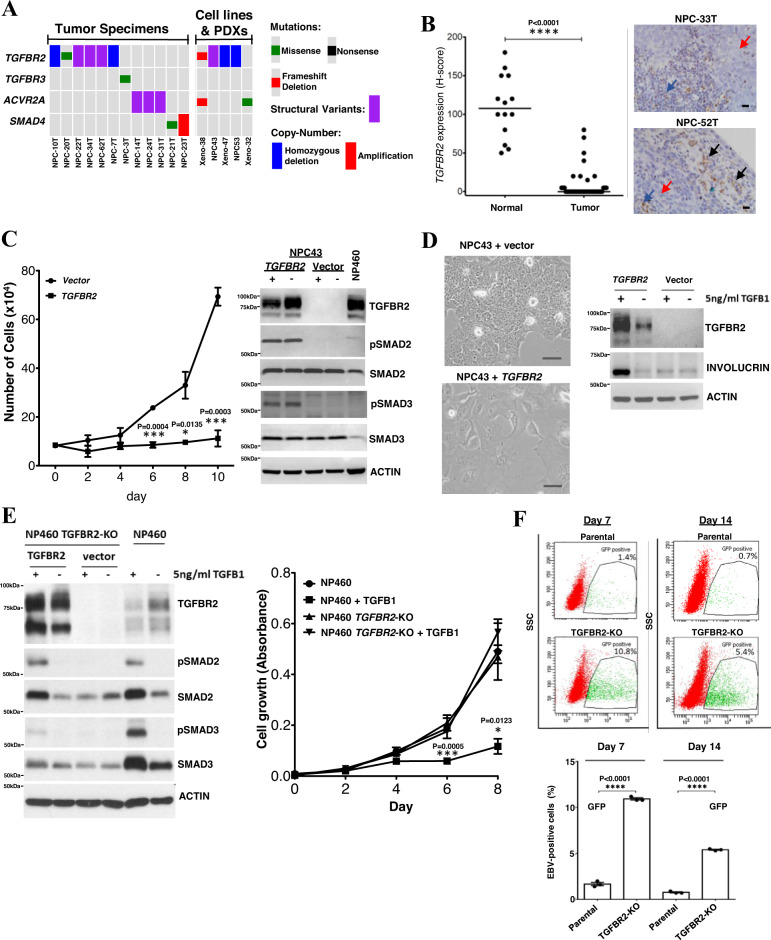
*TGFBR2* loss as a driver event in NPC. **A** Somatic alterations of *TGFBR2* and other genes in TGF-β/SMAD pathway. **B** RISH analysis of *TGFBR2* expression in 41 NPCs and 14 adjacent normal epithelium. Significant downregulation of *TGFBR2* was observed (Unpaired two-tailed *t* test, ****p < 0.0001, mean values of the data are presented) in NPC. Representative NPC cases (NPC33T, NPC-52T) showing no *TGFBR2* expression in the tumor cells (red arrows) and positive signals in the infiltrating lymphocytes (blue arrows) and normal epithelium (black arrows). Scale bar: 10 μm. **C** Dramatic growth inhibition was found in wild-type *TGFBR2* transfected *TGFBR2*-deleted NPC43 cells (unpaired two-tailed *t* test, *p < 0.05; ***p < 0.0005; data are presented as mean values ± SEM). Western blotting showed pSMAD2 and pSMAD3 overexpression in TGFBR2-expressing NPC43 cells. NP460 with TGFBR2 expression was included as control. Replication: *n* = 3 biologically independent experiments. **D** Re-introduction of wild-type *TGFBR2* induced cell differentiation and morphological changes in NPC43 cells. In TGFBR2-expressing NPC43 cells, the levels of involucrin were increased upon TGF-β1 treatment, as compared with the vector control. Replication: *n* = 3 biologically independent experiments. Similar results were found in the repeated experiments. Scale bar: 50 μm. **E** CRISPR/Cas9-mediated knockout of *TGFBR2* in NP460 (NP460KO) cells resulted in the loss of TGFBR2. No pSmad2 and pSmad3 expression were detected in NP460KO cells following TGF-β1 treatment. Re-expression of wild-type TGFBR2 in NP460KO cells restored its response to TGF-β1 as shown by the induction of pSmad2 and pSmad3. NP460KO cells showed significant growth inhibition when compared with parental NP460 cells after TGF-β1 treatment (unpaired two-tailed *t* test, *p < 0.05; ***p < 0.0005; data are presented as mean values ± SEM). Replication: *n* = 5 biologically independent experiments. **F** The parental NP460 and NP460KO cells were infected with a GFP-tagged recombinant EBV. After EBV infection and flow sorting of EBV-positive cells, significantly higher numbers of EBV-positive cells were maintained in *TGFBR2* knockout NP460KO cells than those in parental cells on both day7 and day 14 (unpaired two-tailed *t* test, ****p < 0.0005, data are presented as mean values ± SEM). Replication: *n* = 3 biologically independent experiments. Source data are provided as a Source Data file.

Dysregulation of TGF-β/SMAD signaling has been shown to initiate cancer formation and disease progression in various human cancers^28,29^. Here our data shows a 24.3% (17/70 tumors) rate of TGF-β/SMAD pathway gene alteration in NPC, targeting *TGFBR2, TGFBR3*, *ACVR2A,* and *SMAD4* (Fig. 5A). It has been shown that *TGFBR2* loss protects EBV-infected NPC cells from autocrine or paracrine TGF-β-mediated cytostasis and differentiation through Smad signaling^28^. In fact, we found *TGFBR2* aberrations in NPC patient tumors, cell lines (NPC43, NPC53) and PDXs (Xeno-32, Xeno-38, Xeno-47) (Fig. 5A). Strikingly, using RISH we observed downregulation of *TGFBR2* in the majority of clinical tumor samples when compared with adjacent histological normal epithelial cells and infiltrating lymphocytes (Fig. 5B). Similar findings were observed in NPC models (Supplementary figure 8A).

*TGFBR2* is a major tumor suppressor residing on chr. 3p, the most frequently deleted chromosomal region in NPC and its precancerous lesions^1,2,30^. To date, however, the role of *TGFBR2* loss in the pathogenesis of NPC remains poorly defined. Here, we demonstrated that ectopic *TGFBR2* expression inhibited cell proliferation and restored phosphorylation of Smad2/3 in EBV-positive NPC43 cells in response to TGF-β stimulation (Fig. 5C and Supplementary figure 8B). Most importantly, the restoration of *TGFBR2* induced differentiation of NPC cells, resulting in marked morphological changes along with the expression of a differentiation marker involucrin (Fig. 5D). In immortalized normal nasopharyngeal epithelial (NPE) cells, NP460, *TGFBR2* knockout by CRISPR resulted in inhibition of TGF-β/SMAD signaling and resistance to TGF-β-induced growth inhibition (Fig. 5E). To examine the effect of disrupting TGF-β/SMAD signaling on the outcome and stability of EBV infection in these immortalized NPE cells, the parental NP460 and *TGFBR2* knockout NP460KO cells were infected with a green fluorescent protein (GFP)-tagged recombinant EBV (Akata strain), and the EBV-positive cells were isolated using FACS and grown for 7 and 14 days as we described previously^31^. This experiment demonstrated that the efficiency of persistent EBV infection was significantly increased in NPE cells with *TGFBR2* inactivation (Fig. 5F and Supplementary figure 8B). Our findings establish *TGFBR2* loss as a direct driver for NPC tumorigenesis, playing a pivotal role in attenuated TGF-β signaling and establishment of EBV latency.

### Genomic aberrations in DNA repair and other oncogenic pathways

**Fig. 6:**
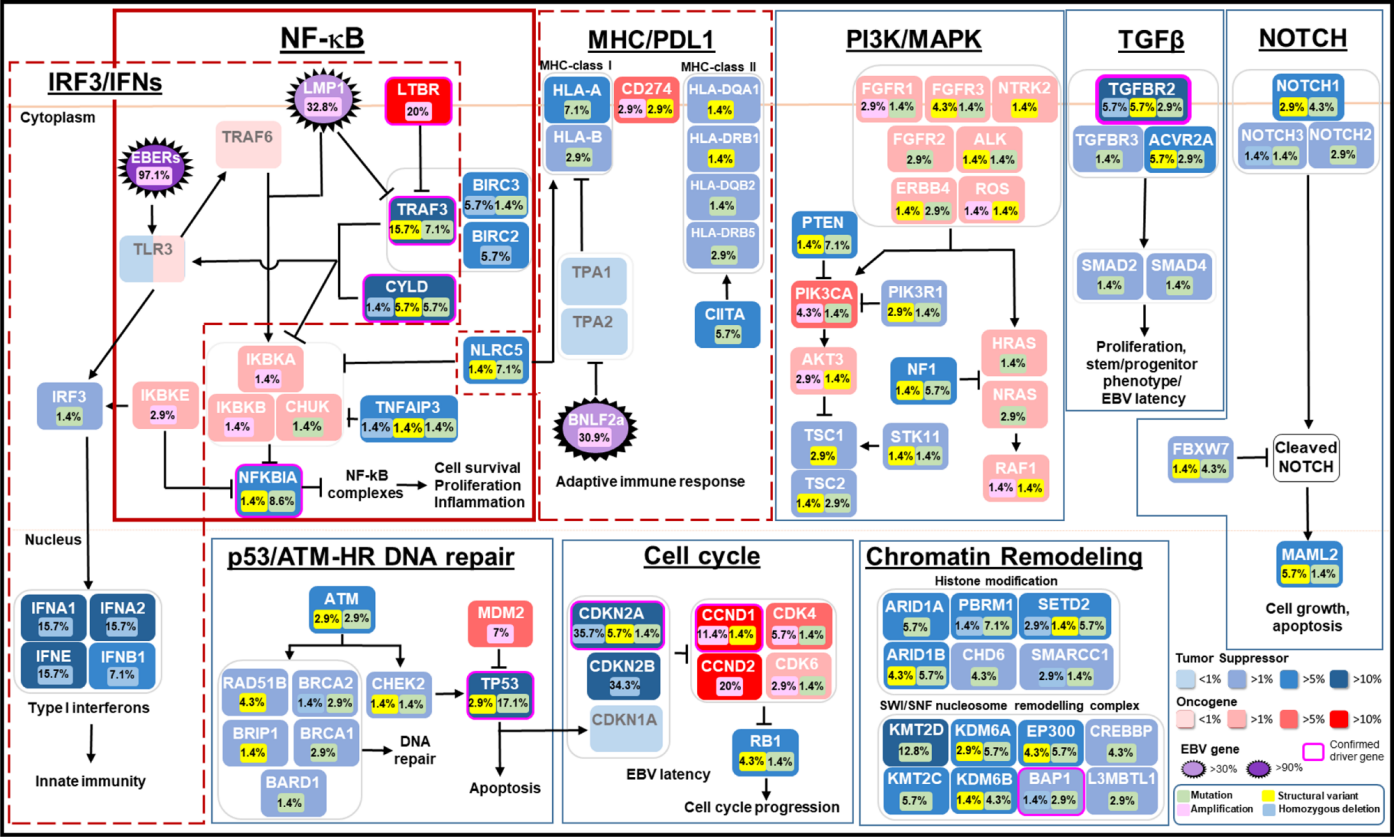
Frequently altered cancer pathways in NPC at the whole-genome level. Multiple genomic aberrations and EBV latent genes alter multiple cancer pathways in NPC.

While somatic alterations of *TP53* and other DNA double-strand break repair genes (e.g., *ATM*, *BARD1, BRCA2, CHECK2*, *RAD51B*) were identified in 20/70 (28.6%) of NPC, we noticed that the HR signature positive tumors were significantly in possession of these mutations (two-sided Fisher’s exact test *p* = 0.044; Figs. 1A and 6). Notably, HR-defect- signature positivity was associated with a significant increase in SV count (two-sided Wilcoxon signed-rank test *p* = 0.016; Supplementary figure 9A). Extensive genome-wide structural alterations (SV count >150) were observed in two NPC clinical specimens (NPC-24T and NPC-38T; Supplementary figure 9A). Both of these highly rearranged tumors had deleterious alterations in an HR pathway member. Specifically, NPC-24T harbored an *ATM-NCOR1* fusion leading to truncation of *ATM* at exon 36, whereas NPC-38T displayed a focal region on chr13q with numerous breakpoints, characteristic of chromothripsis, leading to homozygous loss of *BRCA2*(Supplementary figure 10). The most commonly altered DNA repair gene *TP53*, was associated with a significant reduction in disease-free and overall survival (Supplementary figure 9B). However, patients with mutations in other HR-related gene defects did not experience a different outcome, indicating the *TP53*-altered status is likely associated with disease aggressiveness through mechanisms other than genomic instability, such as cell cycle regulation.

We also observed alterations to the cell cycle machinery alterations affecting 74.3% (52/70) of NPC cases at the whole-genome level. Aside from the aberrations of *TP53*, homozygous deletion and structural alterations of *CDKN2A* ranked at the top (42.9%; 30/70 cases), followed by amplification of *CCND1, CCND2, CDK4,* and *CDK6* (31.4%; 22/70) (Fig. 6, Supplementary figure 11). This is consistent with previously reported roles of the *CDKN2A/CCND1/CDK4* axis in NPC cell growth and persistent EBV latent infection in NPE cells^1,2,31^.

Frequent genomic events were also found within the NOTCH signaling pathway (14/70; 20%) along with members of those affecting the chromatin modification machinery (29/70; 41.4%) (Fig. 6, Supplementary figure 10). In the NOTCH pathway, relatively common alterations in *NOTCH1* (5/70; 7.1%), and *MAML2* (5/70; 7.1%) were noted (Supplementary figure 12). Identification of LOF events, such as inactivating gene rearrangements of *NOTCH1* and *MAML2* support their tumor-suppressive roles in NPC, similar to that described in other head and neck cancers (Supplementary figure 13)^32^. Multiple recurrent aberrations impairing chromatin modification machinery included *ARID1A* (5.7%), *ARID1B* (10%), *CHD6* (4.2%), *KMT2C* (5.7%), *KMT2D* (11.4%), *KDMA6A* (8.6%), and *EP300* (10%) (Fig. 6, Supplementary figure 12). Interestingly, LOF alterations were also commonly found in a cluster on chr 3p21.3 region encompassing genes of the SWI/SNF complex (*BAP1* and *PBRM1*) and chromatin remodeling (*SETD2* and *BAP1*) (11/70, 15.7%) (Supplementary figure 12).

### MTAP deletion is druggable in primary and recurrent NPC

Previous exome studies have shown that there are many potentially druggable targets scattered across the NPC genome^2,8–11,13,14^. Our whole-genome study also revealed hot-spot aberrations of common drug targets including *PIK3CA, EGFR, BRAF, MET,* and *ERBB2*, yet these appeared infrequently (totaling 2.9%, 2/70). Druggable fusion events were identified in 7.1% (5/70) of tumor specimens: *FGFR3-TACC3* (*n* = 2), *NTRK2* fusions (*n* = 1)*, ALK* (*n* = 1), and *ROS1* (*n* = 1). In the case of *NTRK* and *ROS1* fusions, the FDA-approved pan-cancer drugs, larotrectinib, and entrectinib are readily available for clinical treatment in these subsets of NPC patients. Recently in classic Hodgkin lymphoma, a 9p24.1 amplification involving *CD274* (*PD-L1*) and *PDCD1LG2* (*PD-L2*) was shown to predict response to nivolumab outcomes^33^. As a target for immunotherapy, *PD-L1* displayed structural alterations in only 5.7% (4/61) of NPC cases (Fig. 4). Amidst the positive clinical trial results of anti-PD-1 immunotherapy in NPC with 20–34% overall response rates, it would be important to determine if *PD-L1* genomic aberrations can predict outcomes for PD-1/PD-L1 targeting in clinical settings^34–36^. In a recent phase II clinical study of PD-1 inhibitor for recurrent and metastatic NPC, Wang et al. identified a potential association of genomic amplification in 11q13 regions and *ETV6* alterations with poor response^14^. Thus, comprehensive evaluation of both viral factors and somatic alterations targeting host immunity in these clinical trials may help to identify patients who may respond to immunotherapies.

**Fig. 7:**
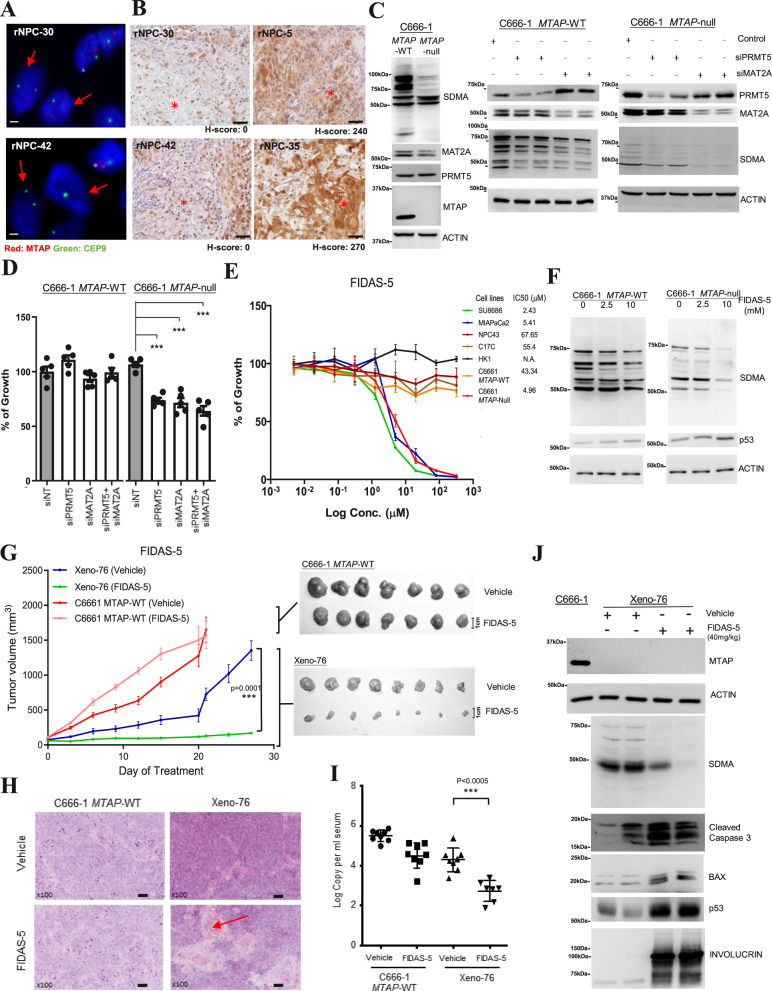
*MTAP* deletion is pharmacologically vulnerable to MAT2A inhibition. **A** FISH analysis detected homozygous deletion of *MTAP* in two NPC tumors (rNPC-30, rNPC-42). Scale bar: 1 μm. **B** Loss of MTAP expression was confirmed in *MTAP*-deleted NPC tumors (rNPC-30, rNPC-42) by IHC. In two NPC tumors without *MTAP* deletion, rNPC-5 and rNPC-35, representative images of MTAP expression were shown. Scale bar: 20 μm. Similar results were found in *n* = 2 independent FISH and IHC experiments. **C** Loss of MTAP expression and reduced SDMA in *MTAP*-null C666-1 cells. Knockdown of *MAT2A* and *PRMT5* by siRNAs repressed SDMA in *MTAP*-null C666-1 cells. Similar effects were observed in *n* = 3 biologically independent experiments. **D** Knockdown of *MAT2A* and *PRMT5* significantly inhibited cell growth of *MTAP*-null C666-1 cells. (unpaired two-tailed *t* test, ***p < 0.0005; data: mean values ± SEM; *n* = 5 biologically independent experiments). **E** FIDAS-5 inhibited the cell growth in *MTAP*-null C666-1 cells, but not parental C666-1 and *MTAP-*intact NPC cells (NPC43, C17C, HK1). The *MTAP*-deleted SU8686 and MIAPaCa2 cells were included as controls. IC50 of each cell line is shown (Data: mean values ± SEM). Replication: *n* = 5 biologically independent experiments. **F** Reduced SDMA and increased p53 expression were observed in FIDAS-5 treated *MTAP*-null C666-1. Similar findings were detected in *n* = 3 biologically independent experiments. **G** Marked in vivo inhibition of *MTAP*-deleted Xeno-76 by FIDAS-5. Growth curves and harvested tumors of C666-1 (*MTAP*-WT) (*n* = 8) and Xeno-76 (*n* = 7) in nude mice treated with vehicle or FIDAS-5 are shown (unpaired ANOVA test, ***p = 0.0001, data: mean values ± SEM). **H** The morphological changes (cell differentiation and keratin production (red arrow)) are illustrated in representative H&E-stained tissue sections of FIDAS-5 treated Xeno-76. Scale bar: 50 μm. **I** Circulating EBV DNA copies were significantly reduced in the Xeno-76 mice after FIDAS-5 treatment. (Unpaired two-tailed *t* test, ***p < 0.0005; data: mean values ± SEM). The blood samples examined in each treatment group of Xeno-76 and C666-1 MTAP-WT are *n* = 8 and *n* = 7, respectively. **J** FIDAS-5 treated Xeno-76 showed reduced SDMA, and increased cleaved caspase 3, BAX, p53, and involucrin expression. Similar effects were shown in *n* = 3 biologically independent experiments. Source data are provided as a Source Data file.

Strikingly, an emerging drug target, *MTAP* (*methylthioadenosine phosphorylase*) was identified as a commonly deleted gene by WGS, accounting for 34% (24/70) of NPC cases. MTAP is a key enzyme for polyamine metabolism and salvaging of adenine and methionine. Studies have revealed the pharmacologic vulnerability of *MTAP*-deficient tumors through targeting the MAT2A/PRMT5 axis^37–40^. *MTAP* resides next to *CDKN2A* on chr. 9p21.3 and is often co-deleted with *CDKN2A*, a frequently deleted tumor suppressor in human cancers. In fact, homozygous deletions of *MTAP* and *CDKN2A* are almost in complete overlap in our NPC cohort and several PDXs including xeno-76 (Fig. 2). In an independent cohort of recurrent NPC, FISH analysis concluded that 32% (16/50) of aggressive tumors harbored *MTAP* homozygous deletion (Fig. 7A, supplementary data 6). The loss of MTAP expression was confirmed in these cases by IHC staining (Fig. 7B and supplementary figure 14A). In addition, the co-deletion of *MTAP* and *CDKN2A* were also confirmed in 7–16.7% of NPC samples in previous whole-exome and WGS studies (Supplementary figure 14B)^12,14^.

We further examined the responsiveness of *MTAP-*deficient NPC to MAT2A inhibition in vitro and in vivo. *MTAP*-null C666-1 cells were generated by CRISPR and subjected to treatment with *MAT2A* siRNA, *PRMT5* siRNA and chemical inhibitor treatments (Fig. 7C–F). As shown in Fig. 7C, the level of symmetric dimethylarginine (SDMA) was markedly reduced in *MTAP*-null C666-1 cells in response to *MAT2A* and *PRMT5* siRNA treatments, indicating significant inhibition of PRMT5 activity. Knockdown of *MAT2A* and *PRMT5* significantly suppressed the growth of *MTAP*-null C666-1, but not that of the parental *MTAP-*WT C666-1 cells (Fig. 7D). Importantly, *MTAP*-null C666-1 cells demonstrated heightened sensitivity (~8.7-fold more sensitive) to the MAT2A inhibitor, FIDAS-5, compared with *MTAP*-wild-type NPC cell lines (Fig. 7E). FIDAS-5 treatment resulted in a reduction in the level of SDMA, accompanied by p53 expression in *MTAP*-null C666-1 cells (Fig. 7F). In vivo, FIDAS-5 elicited marked antitumor activity in an *MTAP*-deficient NPC PDX, Xeno-76 while no such antitumor effect was observed for *MTAP*-WT C666-1 tumors (Fig. 7G, H). In Xeno-76-transplanted mice treated with FIDAS-5, the level of circulating EBV DNA was significantly reduced, associated with an obvious reduction in tumor burden (Fig. 7I). Marked suppression of SDMA was accompanied by the increase of cleaved caspase 3, p53, BAX, and involucrin in the *MTAP*-deficient Xeno-76 tumors (Fig. 7J). At last, FIDAS-5 treatment resulted in the occurrence of tumor cells with keratinization phenotype and involucrin expression in Xeno-76, but not in *MTAP*-WT C666-1 tumors (Fig. 7H and Supplementary figure 14C). Here, we documented the therapeutic vulnerability of *MTAP-*deficient NPC by a MAT2A inhibitor. This precision strategy warrants future assessments in clinical studies, which might well impact a large subset of NPC patients.

## Discussion

The present WGS study establishes a comprehensive whole-genome landscape of a large NPC cohort (*n* = 70), which can serve as a useful resource for the broader community. In addition to genome-wide mutations, rearrangements, and accurate copy number (CN) changes our study incorporates EBV viral gene expression, revealing multiple important insights into NPC tumorigenesis further establishing the interrelated roles for both host somatic alterations and EBV gene expression impacting multiple cellular mechanisms, namely NF-κB activation, immune evasion, and persistent infection with EBV^8–11,13,14^.

Firstly, our results show that constitutive activation of the NF-κB inflammatory pathways occurs in as high as 90% of NPC either through somatic alterations or expression of the virus-encoded LMP1 oncogene, implicating aberrant NF-κB activation as a ubiquitous hallmark of this EBV-associated malignancy. The dominance of NF-κB activation in NPC that we have identified is consistent with recent genome-wide CRISPR-based gene knockout screens and functional studies showing that perturbation of the NF-κB pathway significantly inhibited the growth of EBV-positive NPC cells^8,20,41,42^. These studies, in conjunction with our current findings, conclude the essential role of constitutively activated NF-κB signaling in NPC, a feature distinct from the heterogeneous genomic landscapes of other head and neck cancers.

Second, we identified the mutually exclusive involvement of somatic alterations and overexpression of various viral genes (*LMP1*, *BNLF2a*) targeting innate and adaptive immunity in 91.4% of NPCs. This viral and somatic co-selection is the second viral-somatic collaboration we identified herein. This feature likely arises to counteract the inflammatory environment owing to persistent EBV infection, a unique feature of NPC. Under normal circumstances, the abundantly expressed viral RNAs (e.g., *EBER*s) would trigger potent innate immune response via IRF3-activated type I interferon production while the presentation of multiple immunogenic viral antigens (e.g., EBNA1, LMP2) would facilitate the cytotoxic T-cell responses in EBV-infected NPC^2–4,24,25^. Thus, effective evasion from immune responses is believed to play a key role in the tumorigenesis of this virally associated cancer. In essence, our study defined a panel of genomic and viral events interfering with innate and adaptive immune responses in 78.6% and 47.1% of tumors, respectively, uncovering major mechanisms for NPC immune evasion. Notably, we confirmed the overexpression of the viral TAP inhibitor *BNLF2a* in the latent EBV-infected cells of 18.6% of NPC tumors, which could counteract host immune response. The identification of *BNLF2a* expression, *PDL1* alterations, and other somatic alterations impairing the antigen presentation machinery may inform immunotherapy-related biomarkers or strategies in NPC.

Third, WGS revealed consistent loss of expression and frequent somatic LOF alterations of *CDKN2A* and *TGFBR2*, corroborating their key driver roles in NPC development. In addition to its known role in suppressing cell proliferation and differentiation, our study provides evidence supporting a pivotal role for *TGFBR2* inactivation in persistent EBV infection in NPC. Disruption of TGF-β signaling in NPE cells may facilitate the maintenance of the EBV genome via suppression of cellular differentiation^29^. Since deletion of chr. 3p and 9p which harbor the *TGFBR2* and *CDKN2A* loci, respectively, are consistently found in precancerous lesions of NPC, pointing to a central role for *CDKN2A* loss and impaired TGF-β signaling in creating a susceptible NPE cell population capable of supporting EBV latency during early cancer development^1,2,29–31^.

Finally, a combination of genomics and therapeutic investigation demonstrated the pharmacologic vulnerability of *MTAP*-deleted NPC, which accounts for 32–34% of our cohort. *MTAP* is also commonly deleted in glioblastoma (40%), melanoma (25%), and pancreatic adenocarcinoma (25%);^37–40^ unlike NPC however, those cancers are commonly *TP53*-mutated. Since the majority of *MTAP*-deleted NPC harbors wild-type p53, the high levels of p53 induction triggered by MAT2A inhibitor may underlie the potent tumor suppressor activity observed in NPC in contrast to other *MTAP*-deleted but largely *TP53*-mutated cancers. At present, a phase I clinical trial of MAT2A inhibitor AG-270 (NCT03435250) is ongoing for patients with advanced or refractory solid tumors and lymphomas. We believe our findings will encourage future clinical trials examining *MAT2A* inhibitors in *MTAP*-deleted NPC, including recurrent and metastatic diseases, which are often deadly. This precision medicine strategy, together with potential biomarker-driven immunotherapy may ultimately improve treatment outcomes for patients with advanced NPC.

In conclusion, we have established a comprehensive genome landscape of NPC, and greatly enriched our understanding of NPC etiologies and driver events for tumorigenesis. We demonstrate further the interplays between EBV and somatic alterations, which are co-selected during NPC evolution. Most importantly, our findings provide a rational basis for future precision therapy trials potentially impacting >30% of primary, advanced and recurrent NPC, for the classes of MAT2A and PRMT5 inhibitors.

## Methods

### NPC cell lines, PDXs, and patient tumor samples

All 63 NPC tumor tissues (including two specimens from the same donor) were collected by endoscopy or surgery, embedded in optimal cutting temperature compound, and stored at −70 °C. DNA samples extracted from microdissected frozen tissue sections were subjected to WGS. For all patients, corresponding blood samples were collected as normal control. Written patient consents were obtained from all patients in this study according to institutional clinical research approval. The study protocol was approved by the Joint Chinese University of Hong Kong-New Territories East Cluster Clinical Research Ethics Committee at the Chinese University of Hong Kong, Hong Kong SAR. EBV infection status in these tumor samples was confirmed by *EBER* in situ hybridization^12^. The detailed information of the tumors was provided in supplementary data 1. Three NPC cell lines (NPC43, NPC38, NPC53) and four patient-derived xenografts (xeno-23, xeno-32, xeno-47, xeno-76) recently established by us were included for WGS^12^. Among these samples, 15 tumor specimens, 3 cell lines, and 4 xenografts were also included in our previous exome and genome sequencing studies^8,15^. For functional and preclinical studies, the immortalized NPE cells (NP69, NP460) and authenticated EBV-positive cell lines (C666-1, C17C) and PDXs (xeno-2117, C15, C17) established by us and Prof. Pierre Busson were also used^41,43,44^. The establishment of *MTAP*-deleted C666-1 and *TGFBR2* knockout NP460 cells is described in supplementary methods. All NPC and immortalized NPE cell lines used in this studies cell lines were established by our team (KW Lo and SW Tsao) and have been authenticated by STR (short tandem repeat) profiling and *EBER* in situ hybridization^41,43,44^. The cell lines were tested to be negative for mycoplasma contamination by PCR analysis using primer 5′-YGCCTGVGTAGTAYRYWCGC-3′ (MYCO5) and 5′-GCGGTGTGTACAARMCCCGA-3′ (MYCO3). No commonly misidentified cell lines listed in ICLAC were used in this study.

### Whole-genome sequencing

For tumor and normal samples, 1 μg of genomic DNA was subjected to WGS with a standard 100-bp paired-end read using the Illumina HiSeq 2000 platform (Illumina, San Diego) based on the manufacturer’s instructions^8^. Sequencing libraries were constructed with 500 bp insert length. Raw sequence reads were processed and aligned to the hg38 human reference genome using bwa-mem (v0.7.15). Local realignment was performed around indels using GATK (v3.6-0) and duplicates were marked using Picard (v2.6.0). Mean target coverage of 51× and 83× was achieved for the normal and tumor samples, respectively. BAM files are deposited to the European Genome-phenome Archive under the accession EGAS00001004705.

### Variant calling

Somatic SNVs and small insertions and deletions (indels) were detected using four callers and several downstream filters (see Supplementary Methods). Significantly mutated coding genes and non-coding regulatory elements were identified using ActiveDriverWGS (v0.0.1). Coding genes with mutation rates above background were filtered to include those with more than three non-synonymous mutations excluding notorious passengers^19^. Non-coding calls were limited to open chromatin regions (by ATAC-seq in defined regulatory elements; detailed in Supplementary Methods). SVs were called using three callers and merged, validated, and annotated using MAVIS (v1.8.5), and refined using several downstream filters (see Supplementary Methods).

### CN calling

Allele-specific CN profiles were generated using Varscan2 (v2.3.6) and the R (v3.3.0) package, Sequenza (v2.1.0). Significantly amplified and deleted regions were detected using GISTIC (v2.0.23). Gene-specific copy number calls were generated using a custom R script (See Supplementary Methods). Whole-genome duplication was inferred using code described by López S et al.^45^.

### RNA in situ hybridization

Expressions of *BNLF2a*, *LMP1,* and *TGFBR2* transcripts were detected by RNAscope 2.0 RISH assays using *BNLF2a, LMP1,* and *TGFBR2*-specific probes (Advanced Cell Diagnostics, USA) as previously described^15,46^. A RNAscope probe V-HHV4-BNLF2-C2 was used for the detection of *BNLF2a* transcripts while the probes V-HHV4-LMP1 and BA-V-EBV-LMP1-2EJ were used to confirm the presence of LMP1 transcription signals in the NPC specimens. The cases with LMP1 transcripts signals were excluded from *BNLF2a* detection analysis because of the overlap of the *BNLF2a* gene and exon 3 of *LMP1*. *BNLF2a* and *TGFBR2* expression was assessed by assigning a proportion score and an intensity score. The proportion score relates to the percentage of tumor cells with a positive signal (0–100). The intensity of the signal in the tumor cells was scored as 0 for none; 1 for weak, 2 for intermediate, and 3 for strong. The gene expression score was calculated as the product of the proportion and intensity scores, ranging from 0 to 300.

### Immunohistochemical staining

MHC-I, MHC-II, PDL1, LMP1, MTAP protein expressions were determined on formalin-fixed, paraffin-embedded sections by immunohistochemical staining. After de-waxing, the sections were subjected to antigen retrieval and staining by the automated slide processing system BenchMark XT (Ventana Medical System Inc., Tucson, AZ, USA) as previously published^8,35^. The primary antibody used in this study was anti-LMP1 mouse monoclonal antibody (diluted 1:1000, clone CS.1-4, Dako, Agilent Technologies, USA), anti-PDL1 (22C3, Dako, Agilent Technologies, USA), anti-HLA Class I A/B/C (diluted 1:1000, clone ECR8-5, ab7038, Abcam, USA), anti-MHC Class II (diluted 1:2000, clone 6C6, ab55152, Abcam, USA), anti-MTAP (diluted 1:400; 4158, Cell Signaling, USA), anti-Involucrin (diluted 1:100, clone SY5, MA5-11803, Invitrogen, USA) and p53 (diluted 1:100, clone DO-1, SC-126, Santa Cruz, USA).

### Immunoblotting

Total cell lysates extracted from the xenografts and cell lines were separated by sodium dodecyl sulphate–polyacrylamide gel electrophoresis and transferred to a polyvinylidene difluoride membrane for immunoblotting as described^41^. Primary antibodies used in this study were anti-TGFBR2 (diluted 1:1000, clone D-2, sc-17799, Santa Cruz, USA), anti-SMAD2 (diluted 1:1000, clone D43B4, 5339, Cell Signaling, USA), anti-pSMAD2 (diluted 1:1000, clone 138D4, 3108 Cell Signaling, USA), anti-SMAD3 (diluted 1:1000, clone C67H9, 9523, Cell Signaling, USA), anti-pSMAD3 (diluted 1:1000, clone C25A9, 9520, Cell Signaling, USA), anti-Involucrin (diluted 1:1000, clone SY5, MA5-11803, Invitrogen, USA) anti-MTAP (diluted 1:1000, 4158 S, Cell Signaling, USA), anti-PRMT5 (diluted 1:1000, 2252 S, Cell Signaling, USA), anti-MAT2A(diluted 1:10000, clone B-10, Sc-166452, Santa Cruz, USA), anti-SDMA (diluted 1:1000, 13222 S, Cell Signaling, USA), anti-Caspase-3 (Asp175)(diluted 1:1000, clone 5A1E, 9664, Cell Signaling, USA), anti-BAX (diluted: 1000, 2772, Cell Signaling, USA), anti-p53 (diluted 1:1000, clone DO-1, Sc-126, Santa Cruz, USA), anti-Actin (diluted 1:100000, clone13E5, 4967, Cell Signaling, USA).

### Quantitative polymerase chain reaction

Peripheral blood was collected from the treated mice. DNA was extracted from 100 μl serum using QIAamp Blood mini kit (Qiagen) according to manufacturer’s protocol and eluted in 20 μl elution buffer. Circulating EBV DNA was measured by quantitative PCR that amplified a region in the BamH1-W fragment of EBV genome^47^. Concentrations of EBV DNA were calculated using DNA extracted from EBV-positive Namalwa cells as standard and expressed as copies of EBV per ml serum. The primers and their sequences are shown in Supplementary data 7.

### EBV infection of immortalized NPE cells

Immortalized NPE cells were infected with a GFP-tagged recombinant EBV (Akata strain) using an established protocol^31^. After co-culturing of Akata cells and immortalized NPE cells for 48 h, the infected epithelial cells were subjected to fluorescent-activated cell sorting (FACS) using a BD FACSARIA III system (BD biosciences, USA) to count and isolate GFP(EBV)-positive cells. The isolated EBV-positive cells were then cultured for 7 or 14 days. Growth media were replaced every 48 h. At respective time points, cells were harvested and the percentage of EBV-positive cells was determined using the BD FACSARIA III system.

### Cell proliferation assay

The cell proliferation reagent CCK-8 was purchased from Dojindo Molecular Technologies (Rockville, MD, USA). In general, 5 × 10^3^ cells in 100 μl of medium were seeded into 96-well plates in triplicate. The CCK-8 assay was performed as follows. In brief, 10 μl of CCK-8 reagent was added to each well and incubated at 37 °C for 4 h. The absorbance was then measured at 450 nm. Each sample was performed in triplicate. The relative growth rate was calculated by dividing absorbance at indicated time points by the absorbance at day 1 after cell plating.

### In vitro drug study

In general, 5 × 10^3^ cells in 100 μl of medium were seeded into 96-well plates in triplicate overnight and then treated with various concentrations of FIDAS-5 (Merck Millipore, USA) for 144 hrs. At the end of treatment, the medium was refreshed and 10 μl of CCK-8 reagent (Dojindo Molecular Technologies) was added to each well for assay as described above.

### In vivo mouse experiments

Minced tumor tissues of PDX xeno-76 were subcutaneously inoculated into the flanks of 3–4-week-old female athymic mice with a starting weight of ~18–22 g and allowed to grow to ~50 mm^3^. Mice are kept within animal room limits of 20–23 °C and 40–60% humidity. The mice run on a 12 h light/dark cycle that from 7 am to 7 pm. Eight mice per group were used. FIDAS-5 (40 mg per kg) or vehicle (corn oil) was administered via intraperitoneal injection once daily. Mice were weighed and tumors were measured with a caliper every 3 days. When the tumor sizes exceeded 1000 mm^3^, mice were killed, tumor and blood samples were collected for analysis. Tumor volume was calculated by the formula 0.5 × *l* × *w*^2^, where *l* and *w* are tumor length and width, respectively.

All animal care and experimental procedures were approved by the University Animal Experimentation Ethics Committee (AEEC), the Chinese University of Hong Kong. The animal license was obtained from the Hong Kong Government, Department of Health.

### Reporting summary

Further information on research design is available in the Nature Research Reporting Summary linked to this article.

## Supplementary information

Supplementary Information

Descriptions of Additional Supplementary Files

Supplementary data 1

Supplementary data 2

Supplementary data 3

Supplementary data 4

Supplementary data 5

Supplementary data 6

Supplementary data 7

Reporting Summary

## Data Availability

The raw data in the BAM files for the WGS sequencing from this study have been deposited in European Genome-phenome Archive (EGA) under accession number: EGAS00001004705 in the hyperlink. Access to this data set in EGA can be requested from UHN Genomics Data Access Committee (Contact person: Natalie Stickle, Email: natalie.stickle.uhn.ca). Full mutation and copy number calls are included as part of the supplementary information and the remaining data are available from the authors upon request. A publicly available WGS data set from a cohort of 12 NPC samples deposited at the Sequence Read Archive (SRA, https://submit.ncbi.nlm.nih.gov/subs/sra/) was used for validation of *MTAP* deletion in NPC^12^ (10.1093/carcin/bgy108). The accession codes of these NPC and corresponding normal blood samples are: SRR6431671, SRR6377819, SRR6431672, SRR6377820, SRR6431673, SRR6377821, SRR6431674, SRR6377822, SRR6431667, SRR6377823, SRR6431670, SRR6377824, SRR6431677, SRR6377825, SRR6431678, SRR6377826, SRR6431668, SRR6377827, SRR6431669, SRR6377828, SRR6431675, SRR6377829, SRR6431676, SRR6377830. In addition, simple somatic mutations and SV counts of human cancers reported by Campbell et al. 2017 (10.1101/162784) were used for comparison with those detected in NPC samples in this study. The data set is available in web-link: https://www.biorxiv.org/content/10.1101/162784v1.supplementary material. The NFKB1-binding site motif of the significantly mutated non-coding variant (chr2: 10097565 C > T) is available in: http://jaspar.genereg.net/matrix/MA0105.1/. Gencode v26 is available at: https://www.gencodegenes.org/human/release_26.html. Cosmic mutational signatures V2 (https://cancer.sanger.ac.uk/signatures/signatures_v2/) was used for determining the mutational signatures of NPC samples. Igenomes hg38 reference is available at: http://igenomes.illumina.com.s3-website-us-east-1.amazonaws.com/Homo_sapiens/UCSC/hg38/ Homo_sapiens_UCSC_hg38.tar.gz.  Source data are provided with this paper.

## Source data

Source Data
