# Author Correction: Whole-genome profiling of nasopharyngeal carcinoma reveals viral-host co-operation in inflammatory NF-κB activation and immune escape

**DOI:** 10.1038/s41467-022-32156-9

**Published:** 2022-07-27

**Authors:** Jeff P. Bruce, Ka-Fai To, Vivian W. Y. Lui, Grace T. Y. Chung, Yuk-Yu Chan, Chi Man Tsang, Kevin Y. Yip, Brigette B. Y. Ma, John K. S. Woo, Edwin P. Hui, Michael K. F. Mak, Sau-Dan Lee, Chit Chow, Sharmila Velapasamy, Yvonne Y. Y. Or, Pui Kei Siu, Samah El Ghamrasni, Stephenie Prokopec, Man Wu, Johnny S. H. Kwan, Yuchen Liu, Jason Y. K. Chan, C. Andrew van Hasselt, Lawrence S. Young, Christopher W. Dawson, Ian C. Paterson, Lee-Fah Yap, Sai-Wah Tsao, Fei-Fei Liu, Anthony T. C. Chan, Trevor J. Pugh, Kwok-Wai Lo

**Affiliations:** 1 Princess Margaret Cancer Centre, University Health Network, Toronto, ON Canada; 2 Department of Anatomical and Cellular Pathology, The Chinese University of Hong Kong, Hong Kong SAR, China; 3 State Key Laboratory of Translational Oncology, The Chinese University of Hong Kong, Hong Kong SAR, China; 4 School of Biomedical Sciences, The Chinese University of Hong Kong, Hong Kong SAR, China; 5 Department of Computer Science and Engineering, The Chinese University of Hong Kong, Hong Kong SAR, China; 6 Sir Y.K. Pao Centre for Cancer, The Chinese University of Hong Kong, Hong Kong SAR, China; 7 Department of Clinical Oncology, The Chinese University of Hong Kong, Hong Kong SAR, China; 8 Department of Otorhinolaryngology, Head and Neck Surgery, Prince of Wales Hospital, The Chinese University of Hong Kong, Hong Kong SAR, China; 9 Warwick Medical School, University of Warwick, Coventry, UK; 10 Department of Oral & Craniofacial Sciences and Oral Cancer Research and Coordinating Centre, University of Malaya, Kuala Lumpur, Malaysia; 11 School of Biomedical Sciences, Li Ka Shing Faculty of Medicine, The University of Hong Kong, Hong Kong SAR, China; 12 Department of Radiation Oncology, University of Toronto, Toronto, ON Canada; 13 Department of Medical Biophysics, University of Toronto, Toronto, ON Canada; 14 Ontario Institute for Cancer Research, Toronto, ON Canada

**Keywords:** Head and neck cancer, Tumour virus infections, Cancer genomics, Targeted therapies, Tumour-suppressor proteins

**Correction to:** *Nature Communications* 10.1038/s41467-021-24348-6, published online 07 July 2021

In the Acknowledgements section of this article the grant number relating to Ministry of Higher Education Malaysia given for Lee-Fah Yap was incorrectly given as FP006-2019A and should have been FRGS/1/2019/SKK08/UM/02/6. The original article has been corrected.

