# Supplementary material for: Whole-genome profiling of nasopharyngeal carcinoma reveals viral-host co-operation in inflammatory NF-κB activation and immune escape
Source: Nat Commun. 2021 Jul 7;12:4193. doi: 10.1038/s41467-021-24348-6 (PMC8263564; doi:10.1038/s41467-021-24348-6)
Supplement: Supplementary file 1 — Supplementary Information [file 41467_2021_24348_MOESM1_ESM.pdf]

Supplementary Information

Supplementary Figures:

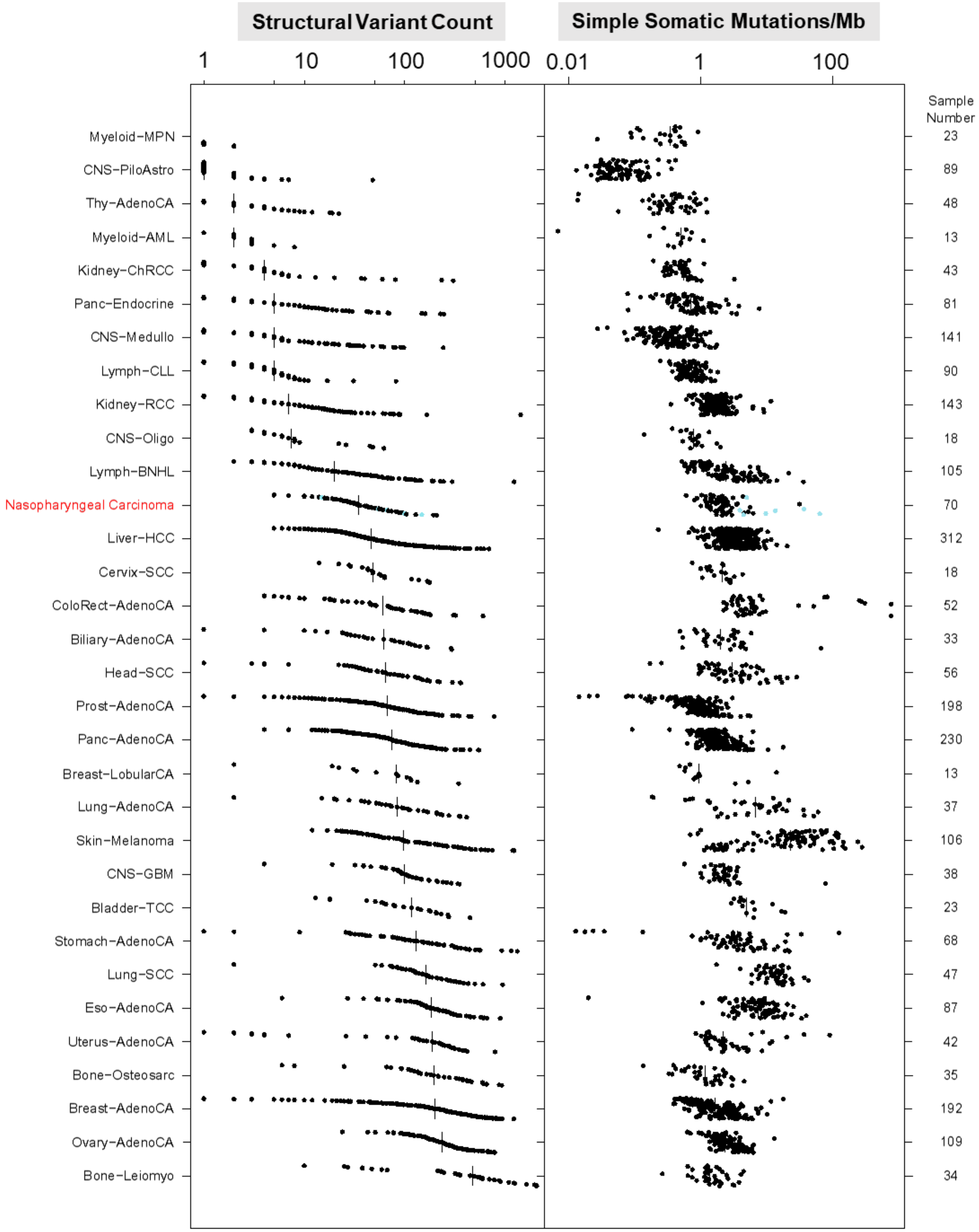

Supplementary figure 1. Simple somatic mutations and structural variant counts of NPC and other human cancers. Black dots: NPC clinical samples; Blue dots: NPC cell lines and PDXs.

Simple somatic mutations and structural variant counts of other human cancers were reported by Campbell et al. (1). The dataset is available in: https://www.biorxiv.org/content/10.1101/162784v1.supplementary-material, (doi:https://doi.org/10.1101/162784).

Significantly mutated non-coding variants chr2:10097565C>T

NFKB1 Motif:

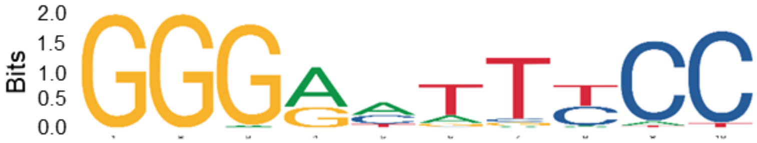

Wild-Type: G G G G C T C C C C C A

Mutant: G G G G C T T C C C C A

Wild-Type:

Target_ID	q-value	E-value
UP00088_1 (Plagl1_primary)	1.03E-02	5.16E-03
NFKB1_DBD	1.59E-01	5.52E-01
MA0105.4 (NFKB1)	1.59E-01	5.52E-01
NFKB2_DBD	1.59E-01	7.06E-01
MA0778.1 (NFKB2)	1.59E-01	7.06E-01
UP00088_2 (Plagl1_secondary)	1.59E-01	7.16E-01
ZNF306_full	1.70E-01	1.02E+00
UP00035_2 (Hic1_secondary)	3.99E-01	2.80E+00
MA0101.1 (REL)	4.50E-01	3.38E+00
ZNF740_full	4.72E-01	4.02E+00

Mutant:

Target_ID	q-value	E-value
NFKB1_DBD	1.87E-02	4.79E-02
MA0105.4 (NFKB1)	1.87E-02	4.79E-02
NFKB2_DBD	1.87E-02	6.03E-02
MA0778.1 (NFKB2)	1.87E-02	6.03E-02
UP00088_1 (Plagl1_primary)	4.38E-02	1.97E-01
MA0101.1 (REL)	4.78E-02	2.39E-01
MA0056.1 (MZF1)	7.49E-02	4.12E-01
ZNF306_full	2.25E-01	1.40E+00
MA0107.1 (RELA)	2.25E-01	1.47E+00
UP00088_2 (Plagl1_secondary)	2.68E-01	2.01E+00

Supplementary figure 2. Comparison between NFKB1 binding site motif of the wild-type and mutant sequences at the non-coding variant locus. Red; significant motif match ($q < 0.05$). The NFKB1 binding site motif is available in: http://jaspar.genereg.net/matrix/MA0105.1/.

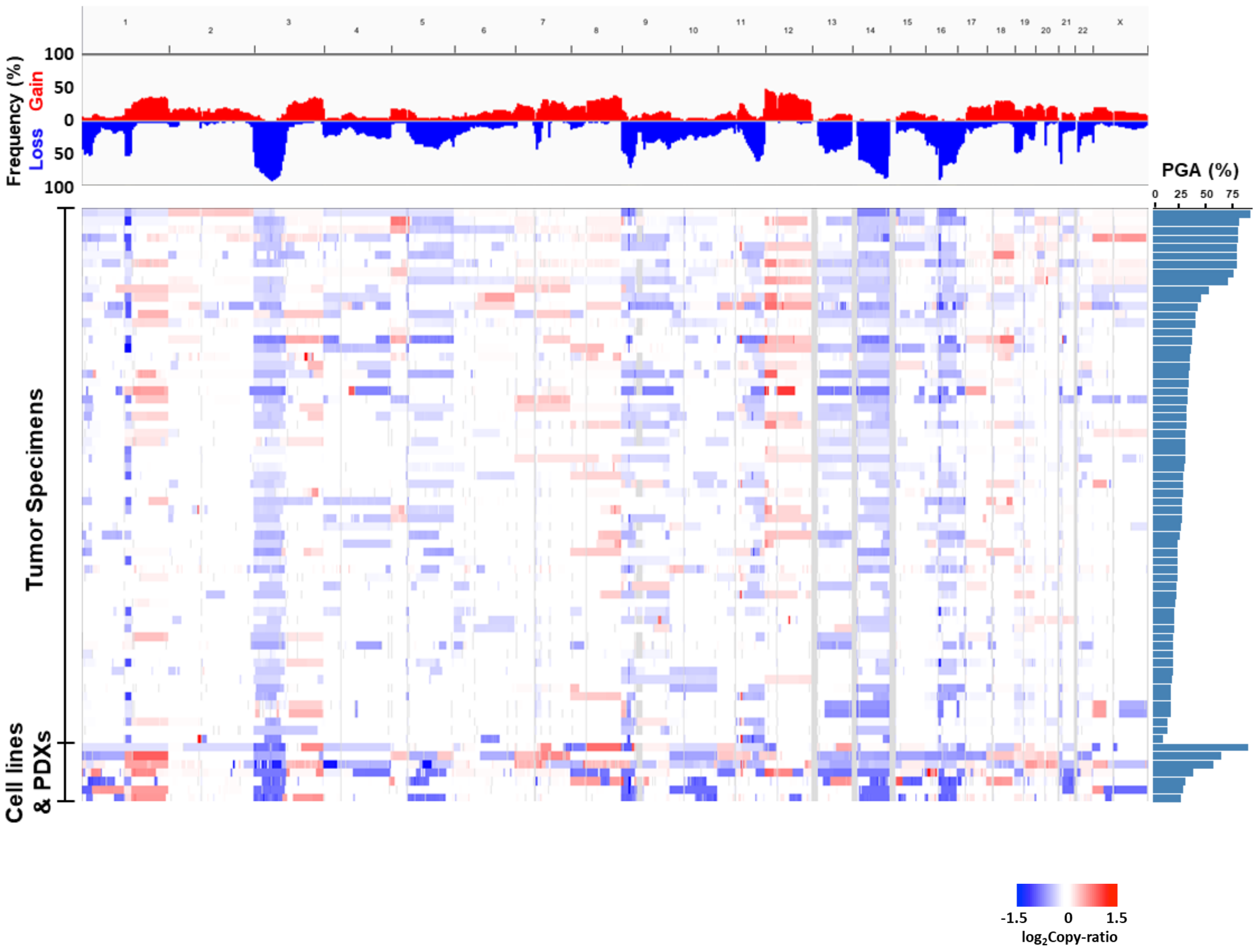

Supplementary figure 3. Global chromosomal gains and losses in 70 NPC tumors. Global chromosomal gains (shown in red) and losses (shown in blue) across 70 NPC genomes showing recurrent arm-level CNV events. Percent genome altered (PGA) of each tumor is also indicated.

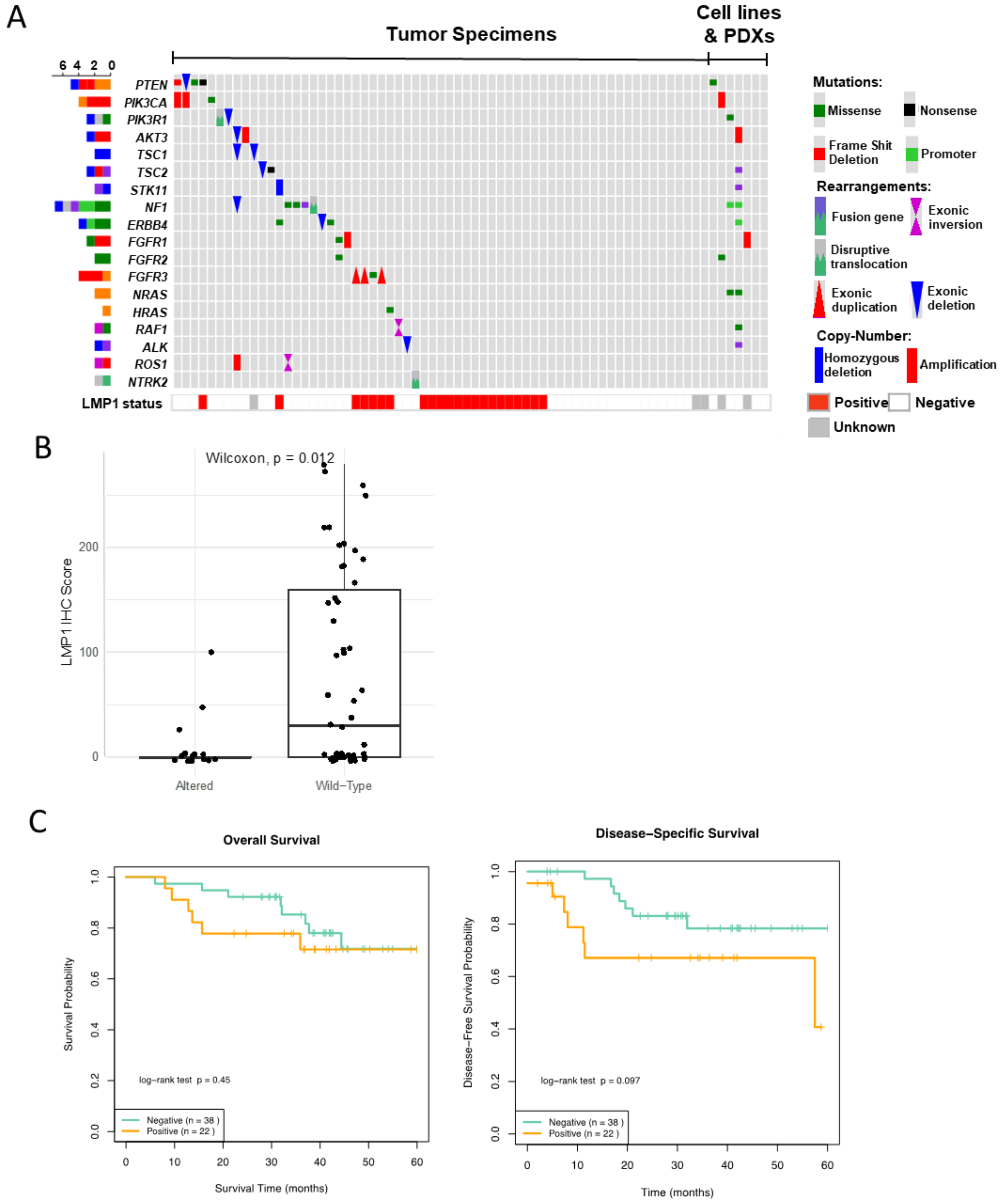

Supplementary figure 4. Somatic alterations in PI3K and RTK signaling pathways. (A) Somatic gene aberrations altering the PI3K and RTK signaling pathways and LMP1 expression in NPC. (B) The correlation of somatic gene alterations of the *PTEN*, *PIK3CA*, *PIK3R1*, *AKT3*, *TSC1*, *TSC2*, *STK11* genes involved in PI3K signaling pathway and LMP1 expression was analyzed by two-sided Wilcoxon signed-rank test. Mutually exclusive relationship between LMP1 expression

and somatic aberrations altering the PI3K signaling pathway is shown ($p = 0.012$). Boxplot of the PI3K-altered (n=22) and PI3K-wild type (n=38) NPC cases is defined as follows: centre upper whisker = min(max(x), Q_3 + 1.5 * IQR), lower whisker = max(min(x), Q_1 – 1.5 * IQR); where IQR = Q_3 – Q_1, the bounds of the box. (C) Correlation of these somatic aberrations altered PI3K signaling pathway and patients' overall and disease-specific survivals (Two-sided Log-rank test $p = 0.45$ and $p=0.097$ respectively). PI3K-altered (Positive: n=22) and PI3K-wild type (Negative: n=38) NPC cases were included in the analysis.

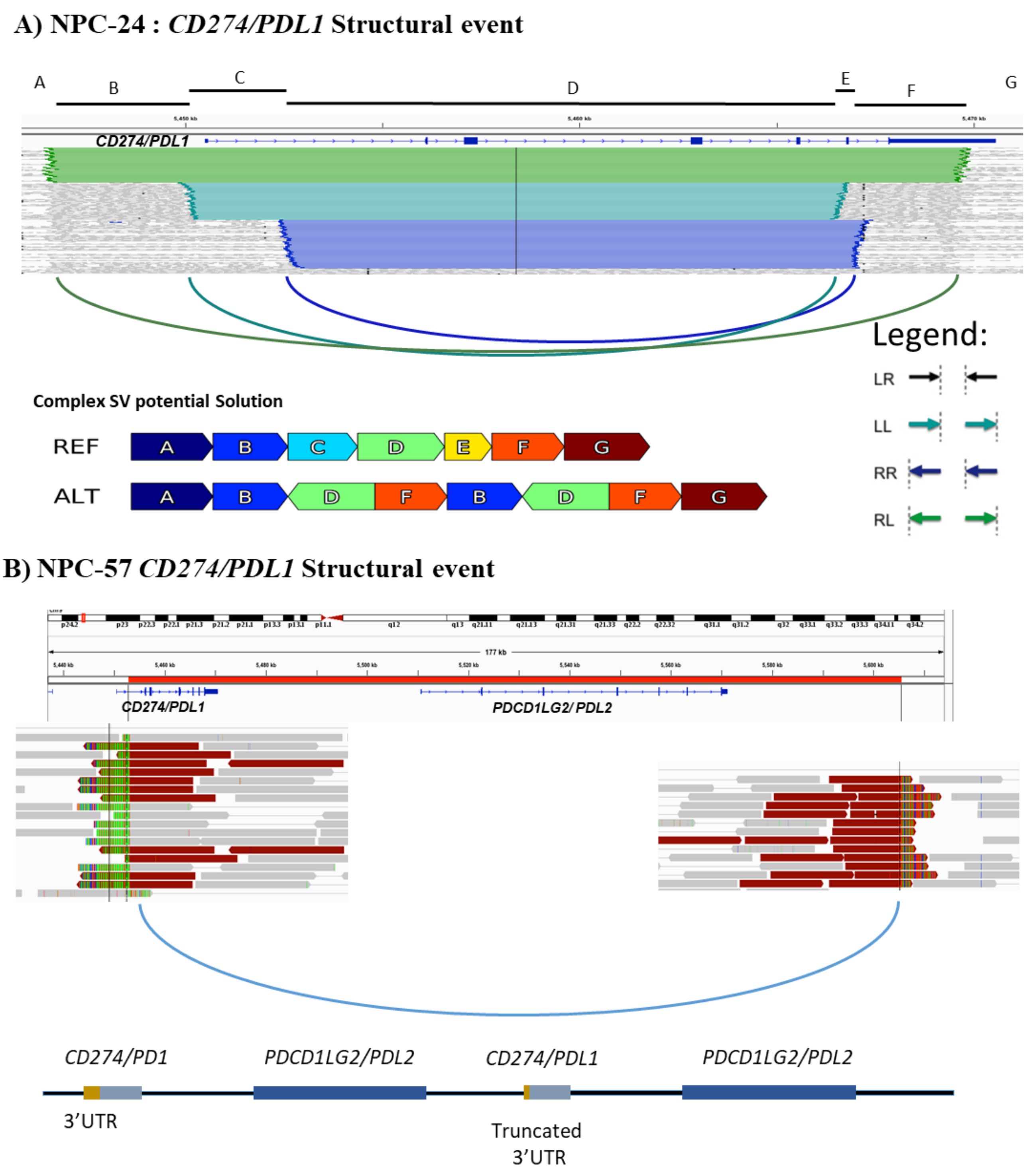

Supplementary figure 5. Structural alterations of *CD274/PDL1* in 2 NPC cases. (A) IGV alignment colored by insert size and pair orientation demonstrating the complex rearrangement involving *CD274/PDL1* in case NPC24T. Panel two shows the putative impact of this complex rearrangement deconvulted by ARC-SV [https://doi.org/10.1101/200170]. (B) IGV alignment demonstrating partial duplication of the *CD273/PDL1* gene in NPC57T. Panel two shows a cartoon depiction of the expected resulting gene structure.

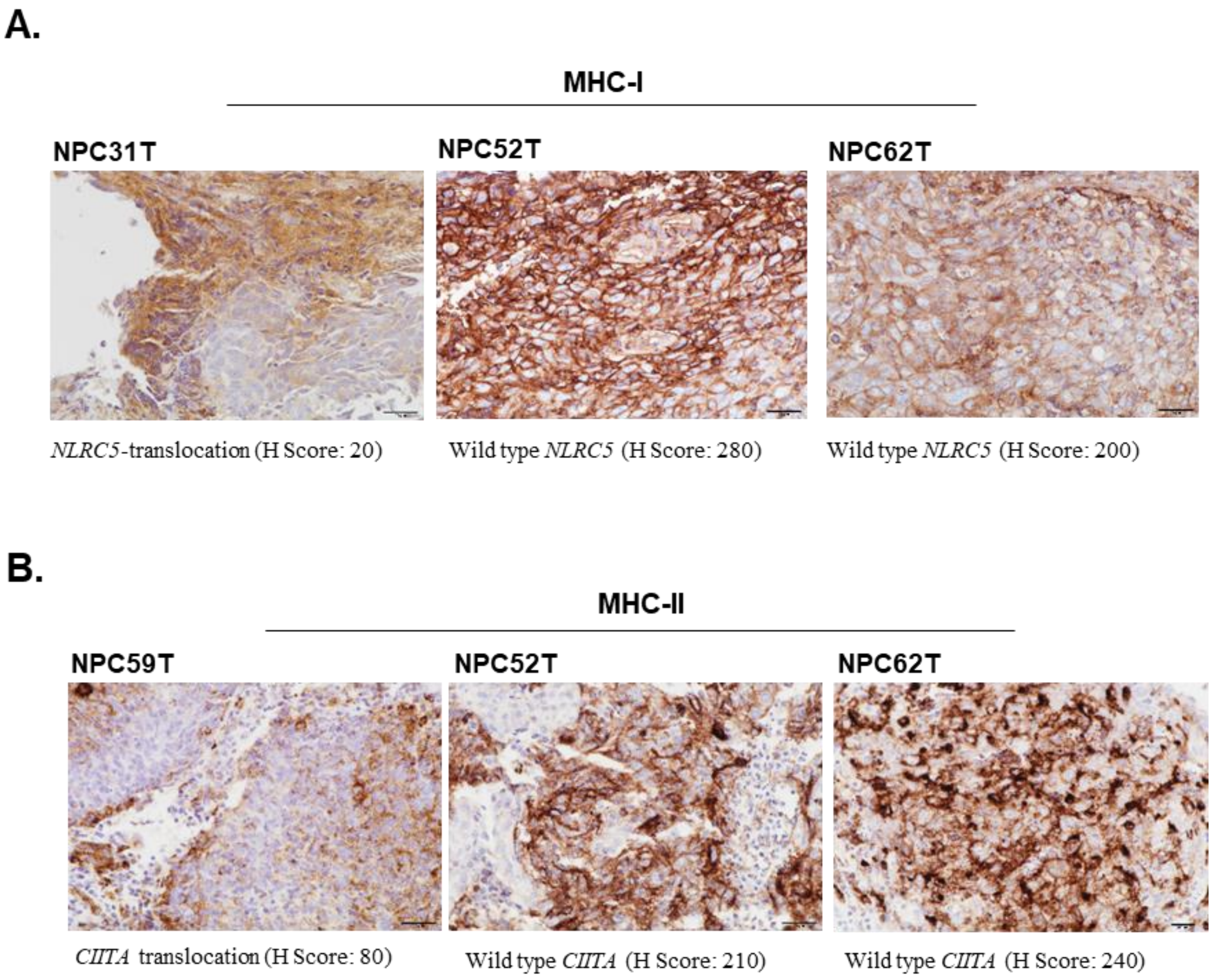

Supplementary figure 6. Loss of MHC-class I and MHC-class II expression are demonstrated in NPC cases with somatic alterations of *NLRC5* and *CIITA* respectively. (A) In a NPC case with *NLRC* translocation (NPC31T), loss of MHC-class I expression was shown. (B) Reduced MHC-class II expression was detected in NPC59T, a NPC case with *CIITA* translocation. Expression of MHC-class I (A) and MHC-class II (B) was shown in two NPC cases with wild type *NLRC5* and *CIITA* genes (NPC53T and NPC62T). Each tumor was subjected to IHC staining twice and similar results were found. Representative images of MHC-class I and MHC-class II expression in the NPC cases with wild type or somatic alterations of *NLRC5* and *CIITA* genes are illustrated. Replication n=2 independent experiments. Scale bar: 20μm.

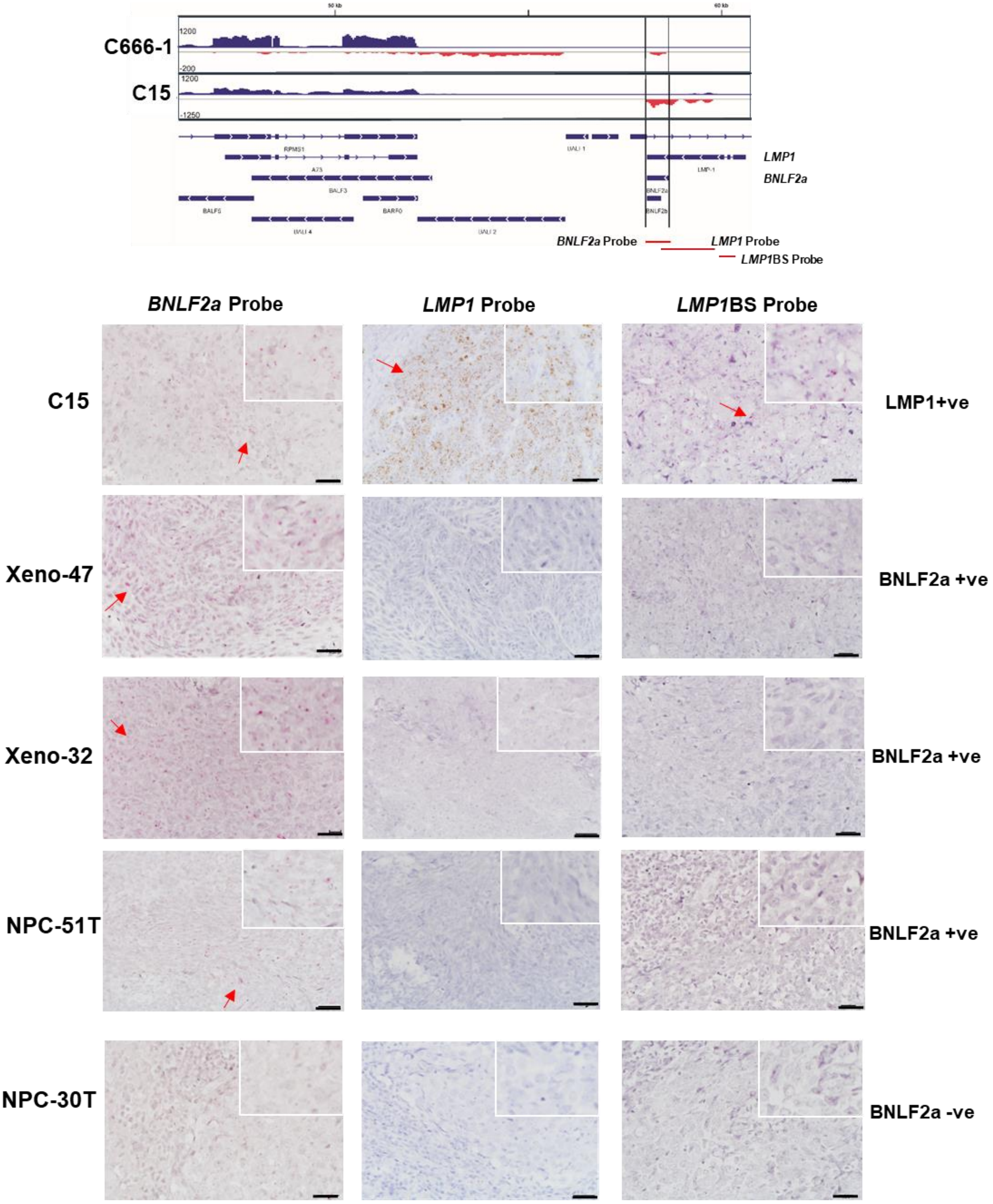

Supplementary figure 7. *BNLF2a* and *LMP1* transcripts in NPC xenografts and tumor samples. In the upper panel, the partial EBV transcription profiles illustrating the expression of *BNLF2a* in C666-1 and C15 (2). The RISH probe targeted regions (*BNLF2a*, *LMP1*, *LMP1*BS) in EBV genome are indicated. Lower panels: representative images of RISH results of *BNLF2a* and *LMP1* in NPC tumors are illustrated. By RISH, specific signals of *BNLF2a* transcripts were shown

in the NPC xenografts (Xeno-47 and Xeno-32) and primary tumor NPC-51T using *BNLF2a* probe, but not the *LMP1* and *LMP1*BS probes targeted 5'region of exon3 and exon junctions of *LMP1* gene respectively. In the LMP1 expressing NPC xenograft C15, signals for *BNLF2a*, *LMP1*, *LMP1*BS probes were detected. Each tumor was subjected to RISH analysis twice and similar results were found. Representative images of the three probes are shown. No signal of both three probes were detected in a *BNLF2a* negative NPC tumor, NPC-30T. Scale Bar: 20μm.

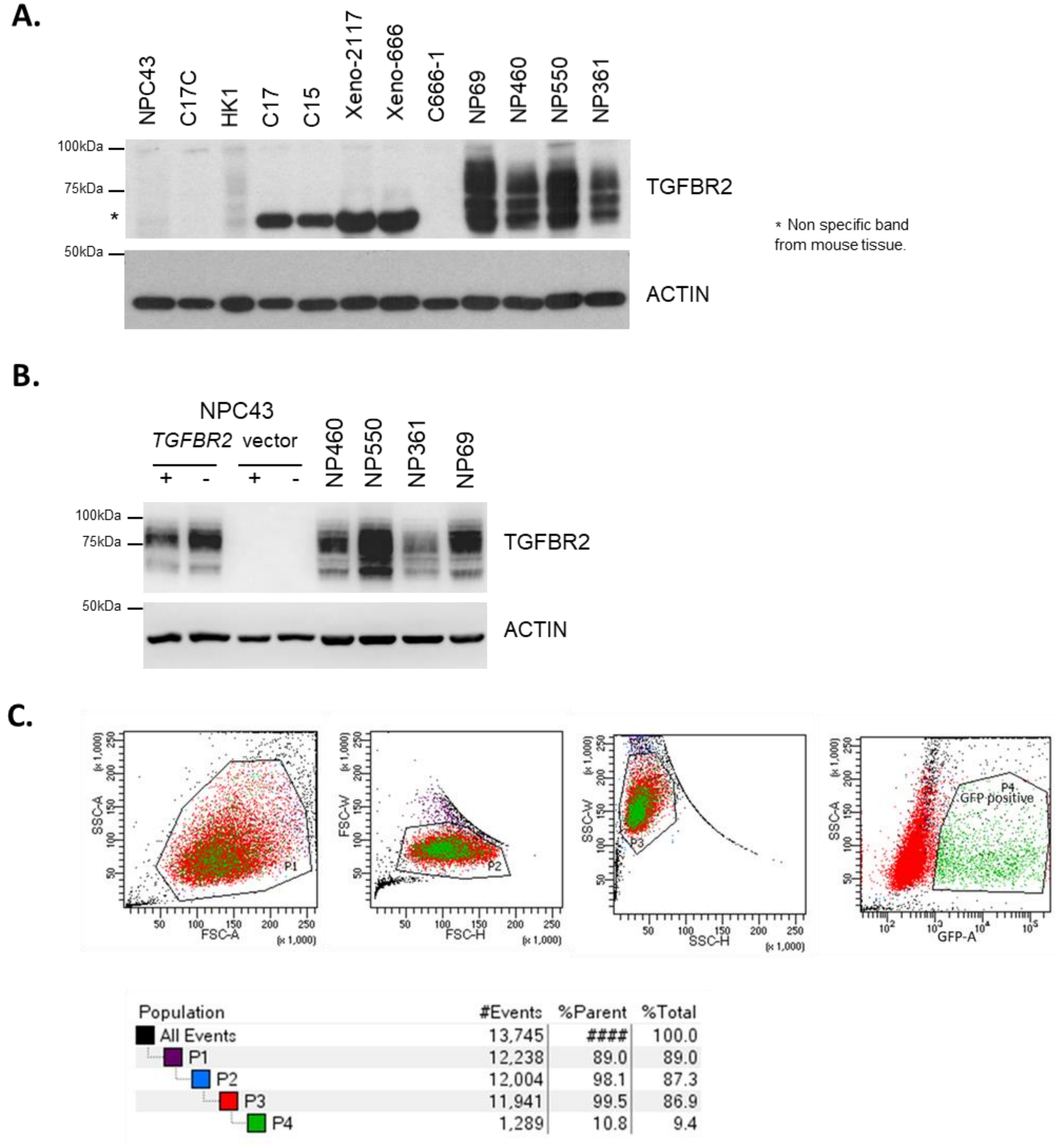

Supplementary figure 8. Expression of TGFBR2 in NPC and immortalized nasopharyngeal epithelial cells. (A) Loss of TGFBR2 protein expression is shown in a panel of EBV-positive NPC cell lines (C666-1, NPC43, C17C) and PDXs (X666, X2117, C15, C17). Weak expression of TGFBR2 was shown in a EBV-negative NPC cell line HK1. As control, expression of TGFBR2 in immortalized nasopharyngeal epithelial cells NP69, NP460, NPC550 and NP361 is indicated. (B) TGFBR2 protein expression in the TGFBR2 transfected NPC43 cells was similar to that in the immortalized normal NP cell lines (NP460, NP550, NP361 and NP69). The protein expression data in these panels is representative of 3 independent experiments with similar results. Source

data are provided as a Source Data file. (C) The gating strategy for FACS sorting and analysis for Figure 5F. For detecting and isolating the EBV-positive cells, the gating strategy is P1: SSC-A vs FSC-A (for identifying cell population), P2: FSC-W vs FSC-H (for identifying single cell population), P3: SSC-W vs SSC-H (for identifying cell population), and P4: SSC-A vs GFP-A (for gating cells with high GFP signals).

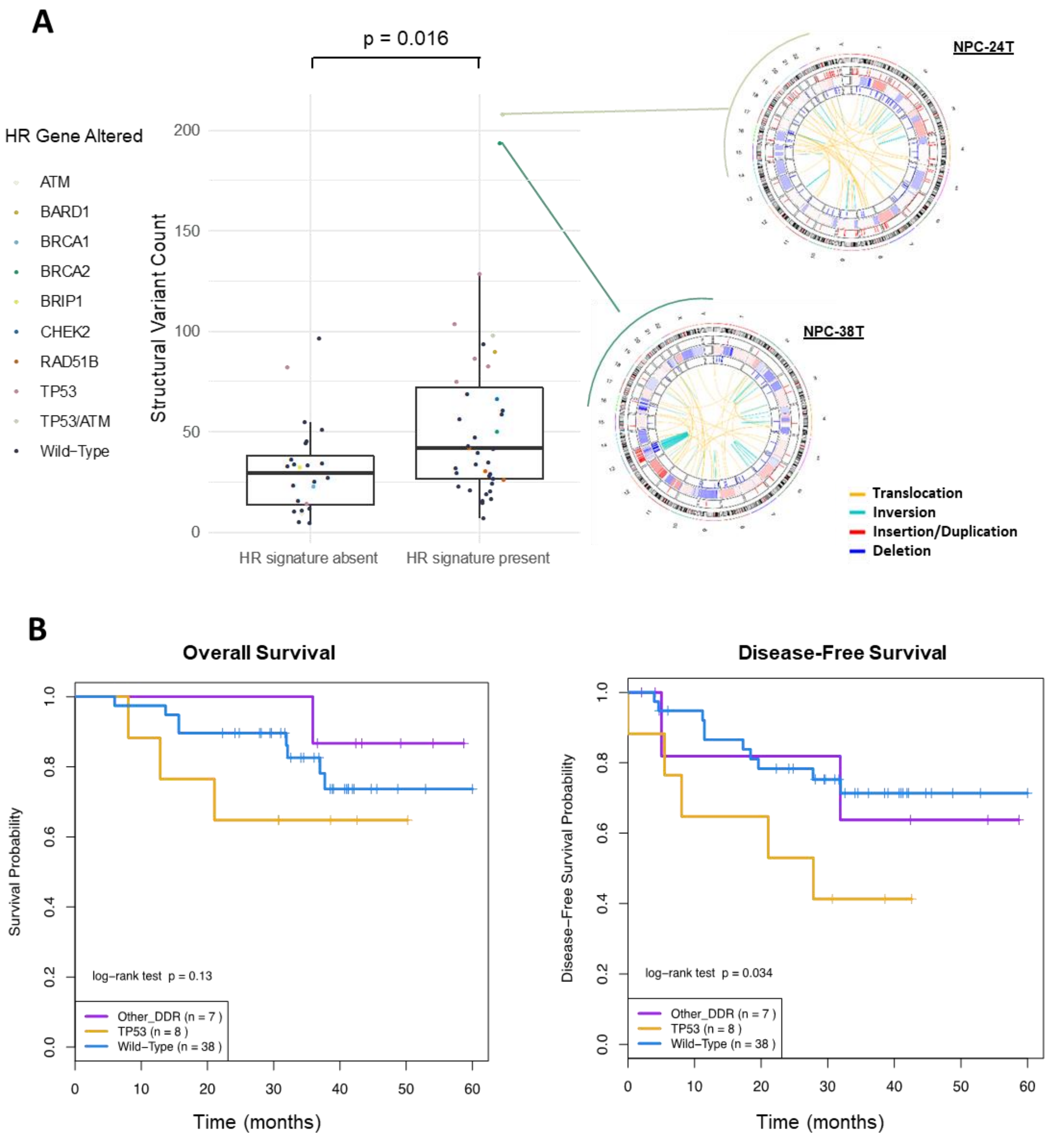

Supplementary figure 9. Somatic alteration of TP53 and double-strand DNA repair (DDR) genes in NPC. (A) The presence of HR-signature is significantly associated with somatic alterations of double-strand DNA repair (DDR) genes in NPC (n=63 tumors, two-sided Wilcoxon signed-rank test p=0.016). Boxplot is defined as follows: centre upper whisker = min(max(x), Q_3 + 1.5 * IQR), lower whisker = max(min(x), Q_1 – 1.5 * IQR); where IQR = Q_3 – Q_1, the bounds of the box. Circos plots of 2 NPCs (NPC-24T and NPC-38T) with the highest SV counts and DDR gene alterations are shown. (B) Somatic alterations of *TP53* gene (n=8), but not other DDR genes (n=7), correlated with poor disease-free survival in NPC patients (two-sided log-rank test p=0.034). Wild type: n=38. No significant correlation of *TP53* gene alterations with overall survival was detected (two-sided log-rank test p=0.13). Source data are provided as a Source Data file.

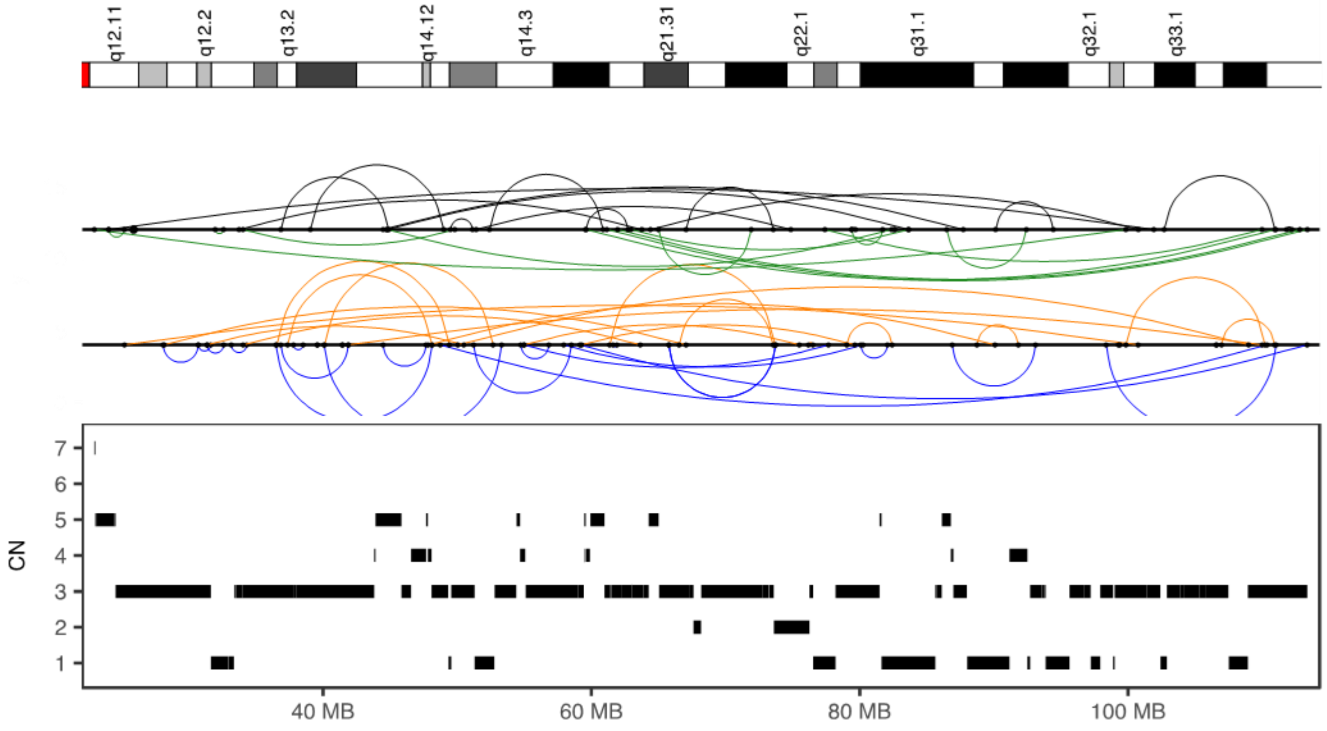

Position	13:19889558–113455691
Total nb. SVs (intrachr. + transl.)	67
SVs in sample	194
Oscillating CN (2 and 3 states)	5
CN segments	83
Pval chr. breakp. enrich.	1
Pval exponential dist. breakpoints	0
Pval fragment joins	0.74
Links with other chrs	

Supplementary figure 10. Chromothripsis of chromosome 13 in NPC. Chromothripsis analysis results using ShatterSeek [https://github.com/parklab/ShatterSeek] of chromosome 13 in NPC-38T depicting a cluster of rearrangements in this area (upper panel) as well as characteristic highly variable segmented copy number (CN) (middle panel). Lower panel indicate the details of structure alterations.

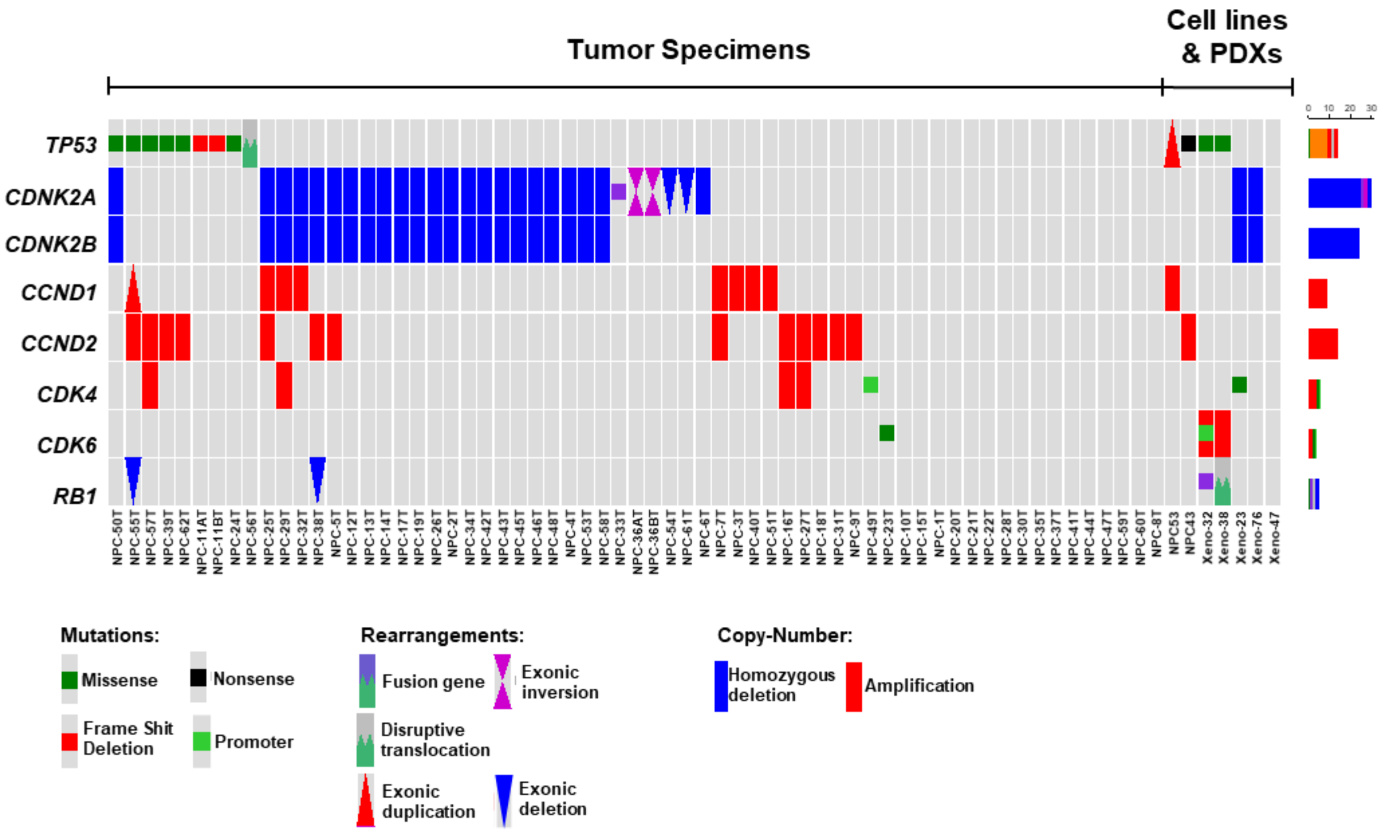

Supplementary figure 11. Somatic gene alterations disrupt cell cycle regulation in NPC. Frequent somatic aberrations impairing cell cycle regulation were detected in NPC tumors.

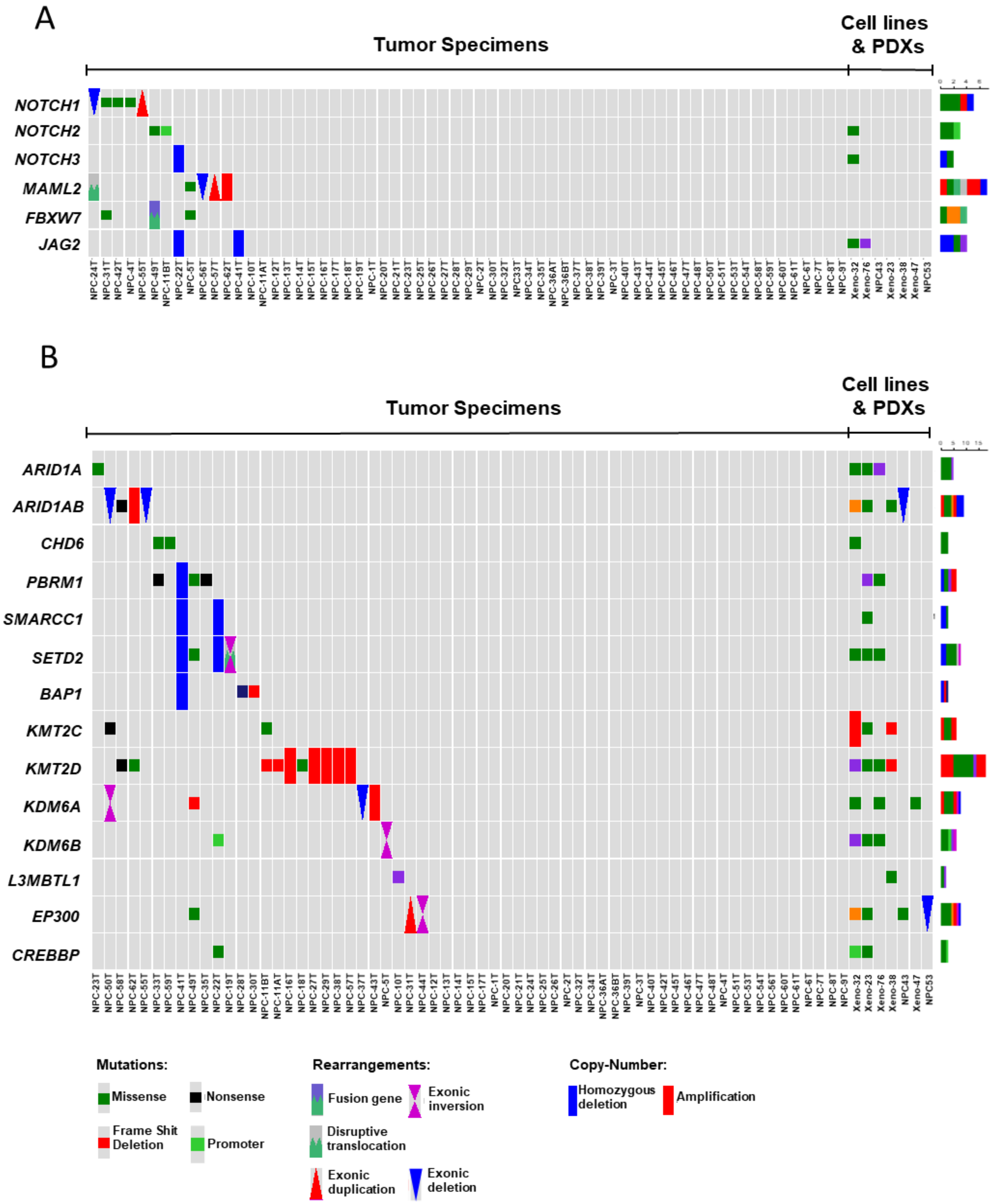

Supplementary figure 12. Somatic gene aberrations altered NOTCH pathways and chromatin modification machinery in NPC. Somatic gene alterations in (A) NOTCH pathways and (B) chromatin modification machinery were commonly detected in 70 NPC tumors.

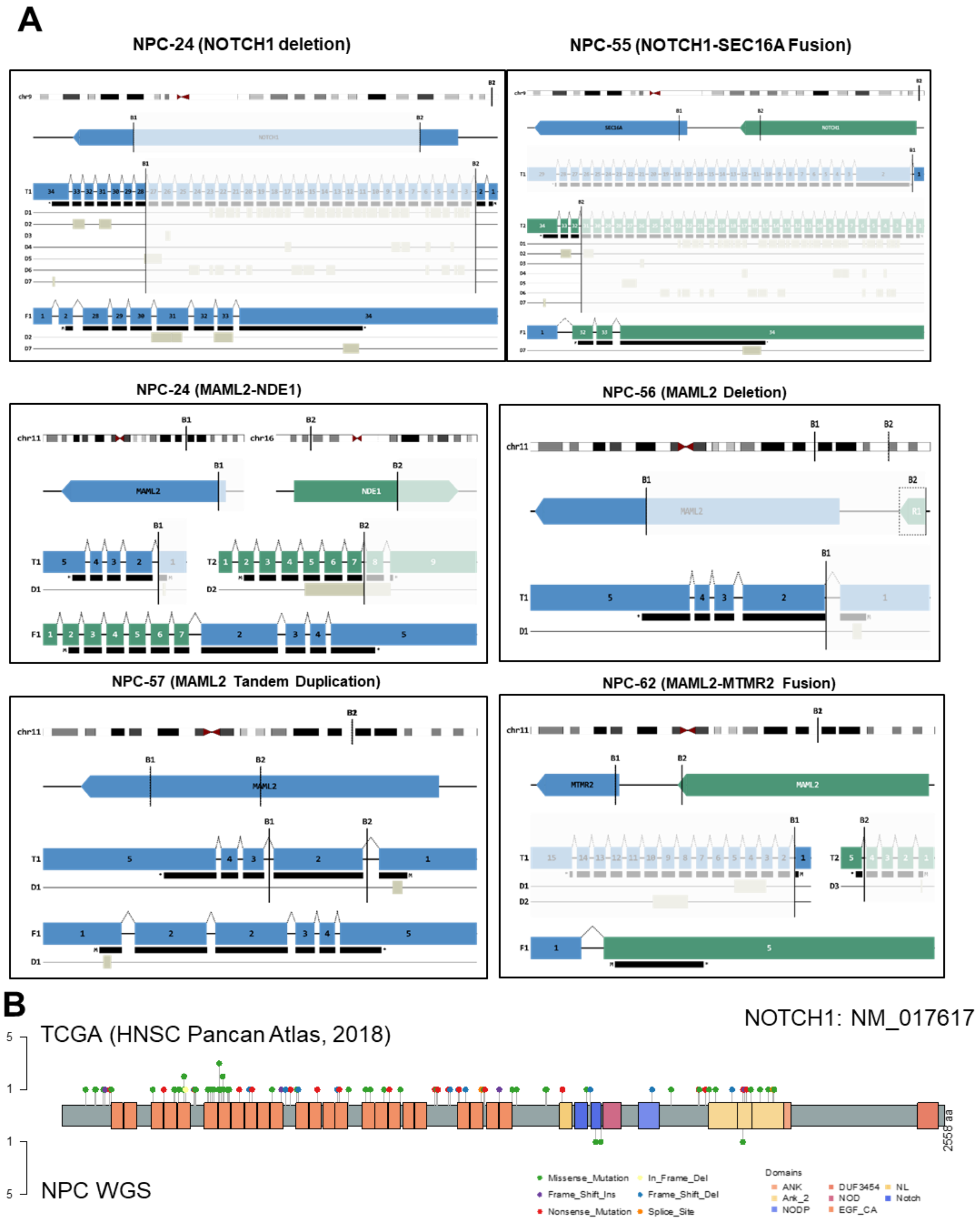

Supplementary figure 13. Somatic gene alterations of *NOTCH1* and *MAML2* identified in NPC. (A) LOF gene rearrangement and deletion of *NOTCH1* and *MAML2* and (B) missense mutations of *NOTCH1* identified in NPC tumors are shown.

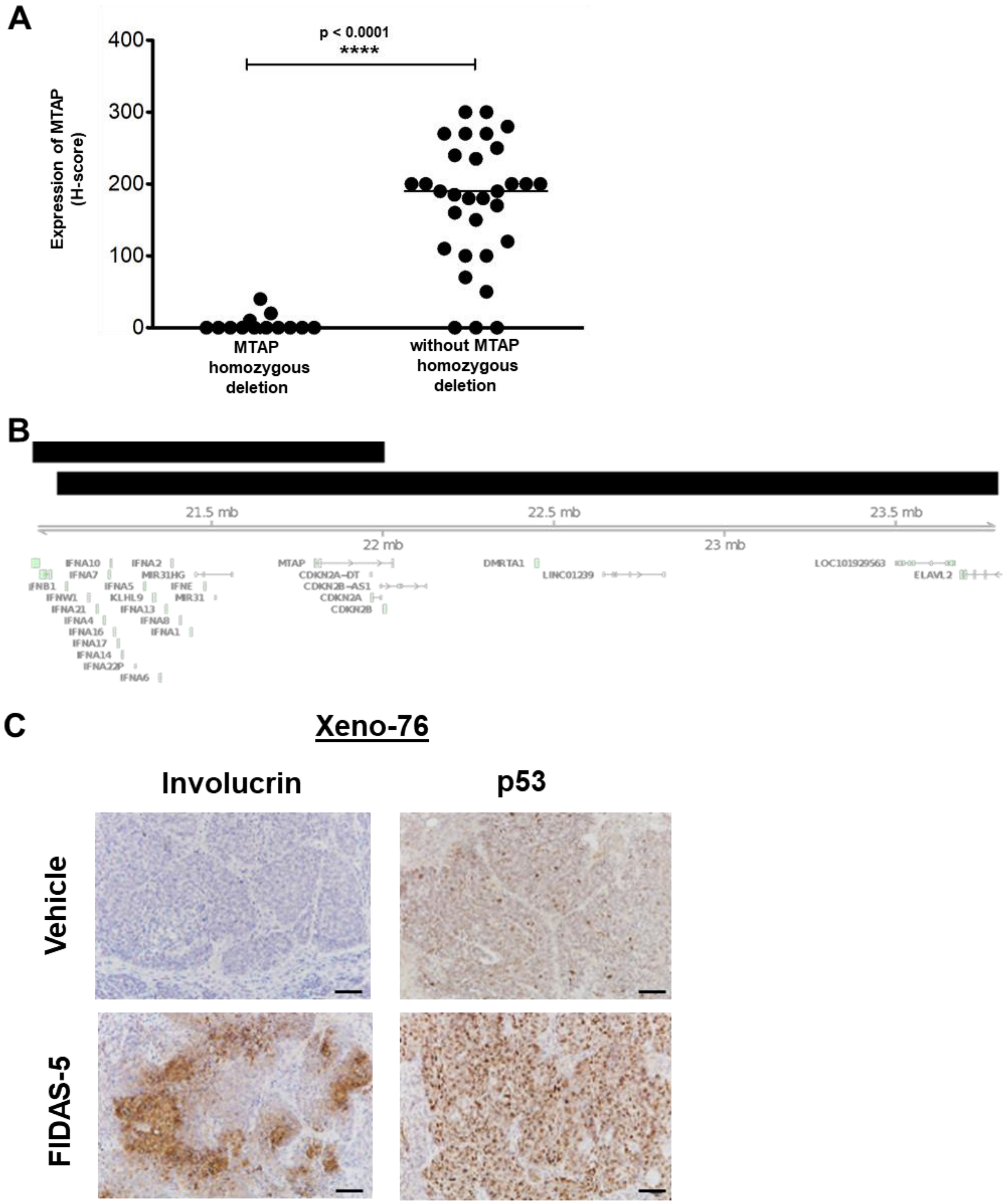

Supplementary figure 14. *MTAP* deletion is a therapeutic target of NPC. (A) Homozygous deletion of *MTAP* are significant correlated with loss of MTAP expression in recurrent NPC tumors (n=50). (Unpaired two tailed t-test, ****p<0.0001, mean values of the data are presented). Immunohistochemistry staining was performed twice in the tumors. Source data are provided as a Source Data file. (B) *MTAP* and *CDKN2A* co-deletions were identified in 2 of 12 NPC cases in a

public available whole-genome sequencing dataset (3). The black bars indicate the deletion regions detected in two NPC tumors. (C) By IHC, increased expression of involucin and p53 were shown in FIDAS-5 treated Xeno-76 tumors. Immunohistochemistry staining was performed twice in the tumors and similar results were observed. Representative images of NPC tumors treated with FIDAS-5 (n=8) or vehicle (n=8) are illustrated. Scale bar: 20μm.

Supplementary Methods:

ATAC-Seq (Assay for Transposase-Accessible Chromatin using sequencing)

50K cells each of two NPC cell lines (C666-1, NPC43) and two immortalized nasopharyngeal cell lines (NP460, NP69) were used for ATAC-seq library preparation as described by Buenrostro et al. (4). Briefly, cells were lysed for 5 min followed by transposase reaction and library amplification. Libraries were then size selected (240-360 bp) and sequenced using 50 bp single reads. Reads were aligned to hg38 with bowtie2 (v2.0.5) using default parameters (5). Aligned reads were then filtered by removing duplicated and mitochondrial reads using samtools (v0.1.18). MACS2 (v 2.0.10) was then use to call open chromatin peaks using the following parameters (6):

macs2 callpeak -t {input.bam} -g hs --keep-dup all -n {sample-name} -B --nomodel --SPMR -q 0.05 --outdir {OutputDir}

The union of peaks in all 4 cell lines was then used for downstream analysis of somatic variants.

Somatic variant calling and filtering

Somatic single nucleotide variants (SNV) and small insertions and deletions were detected using MuTect (v1.1.4), MuTect2 (GATK v3.6-0), Strelka (v 1.0.14), and Varscan2 (v2.3.8). Indels were then left aligned (GATK v3.8) and all somatic calls passing default filters for each caller were then compared using bcftools isec (v 1.2-4) (7-10). Variants called by at least 2 callers were then further filtered to remove calls in repeat regions with poor mapability and variants specific only to PDX samples (11). To identify significantly mutated coding genes and non-coding regulatory elements we used ActiveDriverWGS (v0.0.1) in combination with the following parameters. For coding genes: i) above background mutation rate (FDR < 0.05); ii) greater than 3 non-synonymous mutations; iii) excluding notorious passenger genes (12). For non-coding regions: i) above background mutation rate (FDR < 0.05); ii) within a previously annotated regulatory element (Ensembl regulatory release 94); iii) within open chromatin regions (ATAC-Seq described above).

The proportional contribution of each of the mutation signatures (COSMIC signatures v2) contribution was estimated for each sample using the deconstructSigs (v1.8.0) R package (13-14). The sum-squared error (SSE) of the inferred mutational profiles ranged from 0.03 - 0.15 with a mean SSE of 0.06.

Structural variants (SV) were called using Manta (v1.2.2), DELLY (v 0.7.7) and NovoBreak (v1.1.3) (15-17). SVs from individual callers were merged, annotated and illustrated using MAVIS (v1.8.5) (18). SVs called by at least 2/3 callers were kept and then the following filters were applied i) calls in repeat regions with poor mapability, ii) Identical Breakpoints called in 2+ Normal samples iii) Breakpoints called in >5% of the entire cohort, indicating probably artifacts iv) SVs in genes and loci recurrently called (≥ 2 times) only in PDX samples in order to eliminate potential mouse sequence artifacts (11).

Copy number calling

Allele specific copy-number (CN) profiles were generated using Varscan2 (v2.3.6) and the R (v3.3.0) package, Sequenza (v2.1.0) (10, 19). Significantly amplified and deleted regions were detected using GISTIC2 (v2.0.23) (20). Gene specific copy number calls were generated by overlapping absolute segments with a gene reference (gencode v26). If greater than diploid (2 copies) estimated sample ploidy as called by Sequenza was subtracted from the absolute copy number for each gene to avoid over calling of amplifications. Calls were then made using the following conditionals (in order): Genes with i) absolute value ≥ 4: “amplified”; ii) 2 > absolute value < 4: “gain”; iii) absolute value = 2: neutral iv) 2 > absolute value > 0: “heterozygous loss”; v) absolute value = 0: “homozygous loss”. By copy number calling, the homozygous deletion of *MTAP* has been validated in a WGS dataset from a cohort of 12 NPC samples deposited at the Sequence Read Archive (SRA, https://submit.ncbi.nlm.nih.gov/subs/sra/) (3) (DOI: 10.1093/carcin/bgy108). The accession codes of NPC and corresponding normal blood samples are: SRR6431671, SRR6377819, SRR6431672, SRR6377820, SRR6431673, SRR6377821, SRR6431674, SRR6377822, SRR6431667, SRR6377823, SRR6431670, SRR6377824, SRR6431677, SRR6377825, SRR6431678, SRR6377826, SRR6431668, SRR6377827, SRR6431669, SRR6377828, SRR6431675, SRR6377829, SRR6431676, SRR6377830.

Establishment of *MTAP*-deleted NPC cells and *TGFBR2*-knockout NP cells

MTAP-deleted C666-1 cells were established from the parental C666-1 by CRISPR-Cas9 knockout system (Lipofectamine™ CRISPRMAX™ Cas9 Transfection Reagent, Invitrogen, USA) using

guide RNA (5’-GCCTGGTAGTTGACCTTTGA-3’) targeting exon 4 of *MTAP* gene. NPC460-KO cells were established by *TGFBR2* gene knockout in the telomerase-immortalized nonmalignant human nasopharyngeal epithelial cell line NP460 using CRISPR-Cas9 system and two guide RNAs (gRNA-1, 5’AGTGAGTCACTCGCGCGCA3’ and gRNA-2, 5’GAAGGAAAGTTCAGTTGCA3’) targeting exon 1 of the *TGFBR2* gene. Both *MTAP*-deleted C666-1 cells and NPC460-KO cells were validated by Sanger sequencing and immunoblotting of MTAP and TGFBR2 protein expression, respectively. The NPC cell lines were maintained in RPMI with 10% FBS and supplemented with 1% penicillin/streptomycin.

Supplementary References

1. Campbell, P.J., Getz, G., Stuart, J.M., Korbel, J.O., Stein, L.D. Pan-cancer analysis of whole genomes. bioRxiv 162784; doi: https://doi.org/10.1101/162784 (2017).

2. Lin, W., et al. Establishment and characterization of new tumor xenografts and cancer cell lines from EBV-positive nasopharyngeal carcinoma. Nat. Commun. 9, 4663 (2018).

3. Tu, C., et al. Identification of genomic alterations in nasopharyngeal carcinoma and nasopharyngeal carcinoma-derived Epstein-Barr virus by whole-genome sequencing. Carcinogenesis. 39, 1517-1528 (2018).

4. Buenrostro, J.D., Wu, B., Chang, H.Y. & Greenleaf, W.J. ATAC-seq: a method for assaying chromatin accessibility genome-wide. *Curr Protoc Mol Biol.* **109**, 21.29.1- 21.29.9 (2015).

5. Langmead, B. & Salzberg, S. L. Fast gapped-read alignment with Bowtie 2. *Nat. Methods* **9**, 357-359 (2012).

6. Feng, J., Liu, T., Qin, B., Zhang, Y. & Liu, X.S. Identifying ChIP-seq enrichment using MACS. *Nat Protoc.* **7**, 1728-1740 (2012).

7. Cibulskis, K., et al. Sensitive detection of somatic point mutations in impure and heterogeneous cancer samples. *Nat Biotechnol.* **31**, 213–219 (2013).

8. Van der Auwera, G.A., et al. From FastQ data to high confidence variant calls: the Genome Analysis Toolkit best practices pipeline. *Curr Protoc Bioinformatics.* **43**, 11.10.1-11.10.33 (2013).

9. Saunders, C.T., et al. Strelka: accurate somatic small-variant calling from sequenced tumor-normal sample pairs. *Bioinformatics*. **28**, 1811-1817 (2012).

10. Koboldt, D.C., et al. VarScan 2: somatic mutation and copy number alteration discovery in cancer by exome sequencing. *Genome Res.* **22**, 568-576 (2012).

11. Zhao, X., Emery, S.B., Myers, B., Kidd, J.M. & Mills, R.E. Resolving complex structural genomic rearrangements using a randomized approach. *Genome Bio.* **17**, 126 (2016).

12. Shyr ,C., et al. FLAGS, frequently mutated genes in public exomes. *BMC Med Genomics*. **7**, 64 (2014).

13. Alexandrov, L. B. & Stratton, M. R. Mutational signatures: the patterns of somatic mutations hidden in cancer genomes. *Curr Opin Genet Dev*. **24**, 52–60 (2014).

14. Rosenthal, R., McGranahan, N., Herrero, J., Taylor, B. S. & Swanton, C. deconstructSigs: delineating mutational processes in single tumors distinguishes DNA repair deficiencies and patterns of carcinoma evolution. *Genome Biol.* **17**, 31 (2016).

15. Chen, X., et al. Manta: rapid detection of structural variants and indels for germline and cancer sequencing applications. *Bioinformatics.* **32**, 1220-1222 (2016).

16. Rausch, T., et al. DELLY: structural variant discovery by integrated paired-end and split-read analysis. *Bioinformatics*. **28**, i333-i339 (2012).

17. Chong, Z., et al. novoBreak: local assembly for breakpoint detection in cancer genomes. *Nat. Methods*. **14**, 65-67 (2017).

18. Reisle, C., et al. MAVIS: merging, annotation, validation, and illustration of structural variants. *Bioinformatics*. **35**, 515-517 (2019).

19. Favero, F., et al. Sequenza: allele-specific copy number and mutation profiles from tumor sequencing data. *Ann Oncol.* **26**, 64-70 (2015).

20. Mermel, C.H., et al. GISTIC2.0 facilitates sensitive and confident localization of the targets of focal somatic copy-number alteration in human cancers. *Genome Biol.* **12**, R41 (2011).
