# Supplementary material for: Whole-genome profiling of nasopharyngeal carcinoma reveals viral-host co-operation in inflammatory NF-κB activation and immune escape
Source: Nat Commun. 2021 Jul 7;12:4193. doi: 10.1038/s41467-021-24348-6 (PMC8263564; doi:10.1038/s41467-021-24348-6)
Supplement: Supplementary file 2 — Descriptions of Additional Supplementary Files [file 41467_2021_24348_MOESM2_ESM.pdf]

Descriptions of Additional Supplementary Files

Supplementary Data 1
Description: Clinicopathological details of NPC patients.

Supplementary Data 2
Description: Simple somatic mutations in NPC samples.

Supplementary Data 3
Description: Validation of sequence variants by target-captured sequencing.

Supplementary Data 4
Description: Gene-wise thresholded copy-number calls in NPC samples.

Supplementary Data 5
Description: Structural alterations in NPC samples.

Supplementary Data 6
Description: Clinicopathological details and MTAP status of 50 recurrent NPC tumors.

Supplementary Data 7
Description: Primers and probe for quantitative RT-PCR analysis.
