# Supplementary material for: Whole-genome profiling of nasopharyngeal carcinoma reveals viral-host co-operation in inflammatory NF-κB activation and immune escape
Source: Nat Commun. 2021 Jul 7;12:4193. doi: 10.1038/s41467-021-24348-6 (PMC8263564; doi:10.1038/s41467-021-24348-6)
Supplement: Supplementary file 10 — Reporting Summary [file 41467_2021_24348_MOESM10_ESM.pdf]

nature research

Corresponding author(s): Kwok Wai Lo and Trevor Pugh

Last updated by author(s): Apr 20, 2021

Reporting Summary

Nature Research wishes to improve the reproducibility of the work that we publish. This form provides structure for consistency and transparency in reporting. For further information on Nature Research policies, see our Editorial Policies and the Editorial Policy Checklist.

Statistics

For all statistical analyses, confirm that the following items are present in the figure legend, table legend, main text, or Methods section.

n/a	Confirmed	
☐	☒	The exact sample size (n) for each experimental group/condition, given as a discrete number and unit of measurement
☐	☒	A statement on whether measurements were taken from distinct samples or whether the same sample was measured repeatedly
☐	☒	The statistical test(s) used AND whether they are one- or two-sided<br>*Only common tests should be described solely by name; describe more complex techniques in the Methods section.*
☐	☒	A description of all covariates tested
☐	☒	A description of any assumptions or corrections, such as tests of normality and adjustment for multiple comparisons
☐	☒	A full description of the statistical parameters including central tendency (e.g. means) or other basic estimates (e.g. regression coefficient) AND variation (e.g. standard deviation) or associated estimates of uncertainty (e.g. confidence intervals)
☐	☒	For null hypothesis testing, the test statistic (e.g. F, t, r) with confidence intervals, effect sizes, degrees of freedom and P value noted<br>*Give P values as exact values whenever suitable.*
☒	☐	For Bayesian analysis, information on the choice of priors and Markov chain Monte Carlo settings
☒	☐	For hierarchical and complex designs, identification of the appropriate level for tests and full reporting of outcomes
☒	☐	Estimates of effect sizes (e.g. Cohen's d, Pearson's r), indicating how they were calculated

Our web collection on statistics for biologists contains articles on many of the points above.

Software and code

Policy information about availability of computer code

Data collection	Data were collected in MS Office Excel (different versions)
Data analysis	Publicly available software used for analysis during the current study is described in detail in the main and supplementary methods section. No custom software was designed as part of the current study. The softwares used in this study include: bwa-mem (v0.7.15), GATK (v3.6-0), Picard (v2.6.0), MuTect (v1.1.4), MuTect2 (GATK v3.6-0), Strelka (v 1.0.14), Varscan2 (v2.3.8), ActiveDriverWGS (v0.0.1), Manta (v1.2.2), DELLY (v 0.7.7) and NovoBreak (v1.1.3), ARCSV (v 0.9.6), MAVIS (v1.8.5), Varscan2 (v2.3.6), Sequenza (v2.1.0), GISTIC (v2.0.23), bowtie2 (v2.0.5), samtools (v0.1.18), MACS2 (v 2.0.10), GATK (v3.8), deconstructSigs (v1.8.0), ShatterSeek (v0.4), BD FACSDiva (v8.0.2).

For manuscripts utilizing custom algorithms or software that are central to the research but not yet described in published literature, software must be made available to editors and reviewers. We strongly encourage code deposition in a community repository (e.g. GitHub). See the Nature Research guidelines for submitting code & software for further information.

Data

Policy information about availability of data

All manuscripts must include a data availability statement. This statement should provide the following information, where applicable:
- Accession codes, unique identifiers, or web links for publicly available datasets
- A list of figures that have associated raw data
- A description of any restrictions on data availability

The whole genome sequencing bam files generated during the current study are available in the European Genome-phenome Archive under accession code EGAS00001004705 [https://www.ebi.ac.uk/ega/studies/EGAS00001004705]. All mutation, structural variant and copy-number calls are included in the published article and its supplementary information files.

A complete data availability statement is stated in the manuscript: "WGS data from this study have been deposited in European Genome-phenome Archive (EGA). Under accession number: EGAS00001004705, this WGS dataset is available in the hyperlink: [https://www.ebi.ac.uk/ega/studies/EGAS00001004705]. Full mutation and copy-number calls are included as part of the supplementary information and the remaining data are available from the authors upon request. A publicly available WGS dataset from a cohort of 12 NPC samples deposited at the Sequence Read Archive (SRA, https://submit.ncbi.nlm.nih.gov/subs/sra/) was used for validation of MTAP deletion in NPC (12) (DOI: 10.1093/carcin/bgy108). The accession codes of these NPC and corresponding normal blood samples are: SRR6431671, SRR6377819, SRR6431672, SRR6377820, SRR6431673, SRR6377821, SRR6431674, SRR6377822, SRR6431667, SRR6377823, SRR6431670, SRR6377824, SRR6431677, SRR6377825, SRR6431678, SRR6377826, SRR6431668, SRR6377827, SRR6431669, SRR6377828, SRR6431675, SRR6377829, SRR6431676, SRR6377830. Source data are provided with this paper. In addition, Simple somatic mutations and structural variant counts of human cancers reported by Campbell et al. 2017 (doi: https://doi.org/10.1101/162784) were used for comparison with that detected in NPC samples in this study. The dataset is available in web-link: https://www.biorxiv.org/content/10.1101/162784v1.supplementary-material. The NFKB1 binding site motif of the significantly mutated non-coding variant (chr2: 10097565C>T) is available in: http://jaspar.genereg.net/matrix/MA0105.1/. Gencode v26 is available at: https://www.gencodegenes.org/human/release_26.html. Cosmic mutational signatures V2 (https://cancer.sanger.ac.uk/signatures/signatures_v2/) was used for determination the mutational signatures of NPC samples. Igenomes hg38 reference is available at: http://igenomes.illumina.com.s3-website-us-east-1.amazonaws.com/Homo_sapiens/UCSC/hg38/ Homo_sapiens_UCSC_hg38.tar.gz."

Field-specific reporting

Please select the one below that is the best fit for your research. If you are not sure, read the appropriate sections before making your selection.

☒ Life sciences ☐ Behavioural & social sciences ☐ Ecological, evolutionary & environmental sciences

For a reference copy of the document with all sections, see nature.com/documents/nr-reporting-summary-flat.pdf

Life sciences study design

All studies must disclose on these points even when the disclosure is negative.

Sample size	All available samples of suitable quality were sequenced a part of this study. The sample size of 70 NPCs (63 tumor tissue samples, 4 PDXs and 3 cell lines) represents the largest cohort of NPCs analyzed with whole-genome sequencing to date. For FISH analysis of MTAP deletion, an independent cohort of 50 recurrent NPC patients (49 with local recurrent tumor and 1 with recurrent lymph node metastatic tumor) was included.<br>In our previous whole-exome sequencing study on 78 primary and 33 recurrent NPC with microdissection, we were able to identified the recurrent and critical genetic changes in each group of tumor. Furthermore, a preliminary analysis of 15 WGS data of NPC was also able to detect the newly identify recurrent genetic alterations (MAML2 translocation, TGFBR2 homozgous deletion, LTBR amplification) reported in current WGS study. Similar frequencies of TP53 mutations, TRAF3 alterations were also noted in both WES and WGS studies. The preliminary findings indicated that the cohort of 70 NPC samples in current WGS study are sufficient for identification of the key somatic gene alterations and determination its correlation with EBV oncogene expression.
Data exclusions	No data was excluded.
Replication	In order to improve accuracy and reproducibility of the results we used a multi-caller approach for those simple somatic mutations and structural variants. The multi-caller approach has successfully identified the recurrent somatic gene alterations in 70 NPC samples. To confirm the identified somatic mutations, we also performed a mutation validation experiment using a targeted panel which yield a 96% validation rate of variants at >10% allelic fraction. We also validated several structural variants through Sanger sequencing and fluorescence in-situ hybridization. In this study, the replication of the experiments were indicated in the methods section and Figure legends of the figures and supplementary figures in the revised manuscript. In vitro experiments were repeated at least twice or three times with similar results. In vivo experiments were performed with the indicated number of mice per treatment arm.
Randomization	This study represents a comprehensive overview of the whole genome landscape of NPC as well as functional validation of some key biological findings. Treatment response, prognostic/predictive markers were not the goal of this study and therefore randomization was not necessary. For the in vivo experiments, mice were randomized based upon tumor size into the respective groups as indicated in the manuscript.
Blinding	Treatment response, prognostic/predictive markers were not the goal of this study and therefore blinding was not necessary.

Reporting for specific materials, systems and methods

We require information from authors about some types of materials, experimental systems and methods used in many studies. Here, indicate whether each material, system or method listed is relevant to your study. If you are not sure if a list item applies to your research, read the appropriate section before selecting a response.

Materials & experimental systems

n/a	Involved in the study
	☒ Antibodies
	☒ Eukaryotic cell lines
☒	Palaeontology and archaeology
	☒ Animals and other organisms
	☒ Human research participants
☒	Clinical data
☒	Dual use research of concern

Methods

n/a	Involved in the study
☒	ChIP-seq
	☒ Flow cytometry
☒	MRI-based neuroimaging

Antibodies

Antibodies used

Primary antibodies used in this study were anti-TGFBR2 (D-2, Santa Cruz, USA), anti-SMAD2 (D43B4, Cell Signaling, USA), anti-pSMAD2 (138D4, Cell Signaling, USA), anti-SMAD3 (C67H9, Cell Signaling, USA), anti-pSMAD3 (C25A9, Cell Signaling, USA), anti-Involucrin (SY5, Invitrogen, USA) anti-MTAP (4158S, Cell Signaling, USA), anti-PRMT5 (2252S Cell Signaling, USA), anti-MAT2A (Sc-166452, Santa Cruz, USA), anti-SDMA (13222S Cell Signaling, USA), anti-Caspase-3 (5A1E, Cell Signaling, USA) and anti-BAX (#2772, Cell Signaling, USA), anti-p53 (Sc-126, Santa Cruz, USA), anti-Actin (13E5, Cell Signaling, USA), anti-LMP1 mouse monoclonal antibody (CS.1-4, Dako, Agilent Technologies, USA), anti-PD-L1 antibody (clone 22C3, PD-L1 IHC 22C3; pharmDx assay; Agilent Technologies, USA), anti-HLA Class I A/B/C (ab7038, Abcam, USA), anti-MHC Class-II (ab55152, Abcam, USA).

Validation

All antibodies were validated in the multiple human cell lines as described in the data sheets from the manufacturers.
anti-TGFBR2 (D-2, Santa Cruz, USA) - TGFβ Receptor II/TGFBR2 Antibody (D-2) is a mouse monoclonal IgG2b κ, cited in 27 publications. The antibody was validated in the cell lines n U-698-M, Raji, IMR-32, TF-1 and HT-1080 by Western blot analysis. Selected publications: (1). Wohlfert, E.A., et al. 2004. Resistance to CD4+CD25+ regulatory T cells and TGFβ in Cbl-b-/- mice. J. Immunol. 173: 1059-1065. (2). Tong, J., et al. 2014. TGF-β1 stimulates human Tenon's capsule fibroblast proliferation by miR-200b and its targeting of p27/kip1 and RND3. Invest. Ophthalmol. Vis. Sci. 55: 2747-2756. (3). Monteiro, D.A., et al. 2021. Fluid shear stress generates a unique signaling response by activating multiple TGFβ family type I receptors in osteocytes. FASEB J. 35: e21263.

anti-SMAD2 (D43B4, Cell Signaling, USA) - Smad2 (D43B4) XP® Rabbit mAb #5339 was validated in the HeLa cells and SMAD2 knock-out cells by Western blot analysis. It was cited in 283 publications. Selected publications: (1) Zhang X, Guan T, Yang B, Gu HF, Chi Z. Cell Death Dis. 2020 Jul 17;11(7):544. (2) Jackson-Weaver O, Ungvijanpunya N, Yuan Y, Qian J, Gou Y, Wu J, Shen H, Chen Y, Li M, Richard S, Chai Y, Sucov HM, Xu J. Cell Rep. 2020 Jun 9;31(10):107739. (3) Corbet C, Bastien E, Santiago de Jesus JP, Dierge E, Martherus R, Vander Linden C, Doix B, Degavre C, Guilbaud C, Petit L, Michiels C, Dessy C, Larondelle Y, Feron O. Nat Commun. 2020 Jan 23;11(1):454.

anti-pSMAD2 (138D4, Cell Signaling, USA) - Phospho-Smad2 (Ser465/467) (138D4) Rabbit mAb #3108 was validated in the untreated or TGF-beta treated HeLa and NIH/3T3 cells by Western blot analysis. It was cited in 440 publications. Selected publications: (1) Desbois M, Udyavar AR, Ryner L, Kozlowski C, Guan Y, Dürrbaum M, Lu S, Fortin JP, Koeppen H, Ziai J, Chang CW, Keerthivasan S, Plante M, Bourgon R, Bais C, Hegde P, Daemen A, Turley S, Wang Y. Nat Commun. 2020 Nov 4;11(1):5583. (2) Jackson-Weaver O, Ungvijanpunya N, Yuan Y, Qian J, Gou Y, Wu J, Shen H, Chen Y, Li M, Richard S, Chai Y, Sucov HM, Xu J. Cell Rep. 2020 Jun 9;31(10):107739. (3) Saraswati S, Lietman CD, Li B, Mathew S, Zent R, Young PP. FASEB J. 2020 Jun;34(6):7885-7904.

anti-SMAD3 (C67H9, Cell Signaling, USA) - Smad3 (C67H9) Rabbit mAb #9523 was validated in control HeLa cells and HeLa cells with an apparent in-frame truncation mutation in the gene encoding SMAD3 by Western blot analysis. It was cited in 315 publications. Selected publications: (1) Fan J, Shen W, Lee SR, Mathai AE, Zhang R, Xu G, Gillies MC. Theranostics. 2020 Jun 29;10(18):7956-7973. (2) Jackson-Weaver O, Ungvijanpunya N, Yuan Y, Qian J, Gou Y, Wu J, Shen H, Chen Y, Li M, Richard S, Chai Y, Sucov HM, Xu J. Cell Rep. 2020 Jun 9;31(10):107739. (3) Nguyen DTT, Lu Y, Chu KL, Yang X, Park SM, Choo ZN, Chin CR, Prieto C, Schurer A, Barin E, Savino AM, Gourkanti S, Patel P, Vu LP, Leslie CS, Kharas MG. Nat Commun. 2020 Apr 24;11(1):2026.

anti-pSMAD3 (C25A9, Cell Signaling, USA) - Phospho-Smad3 (Ser423/425) (C25A9) Rabbit mAb #9520 was validated in HT-1080, C2C12, or KNRK cells, untreated (-) or treated with TGF-β (10 ng/ml, 30 min; +) by Western blot analysis. It was cited in 428 publications. Selected publications: (1) Fan J, Shen W, Lee SR, Mathai AE, Zhang R, Xu G, Gillies MC. Theranostics. 2020 Jun 29;10(18):7956-7973. (2) Jackson-Weaver O, Ungvijanpunya N, Yuan Y, Qian J, Gou Y, Wu J, Shen H, Chen Y, Li M, Richard S, Chai Y, Sucov HM, Xu J. Cell Rep. 2020 Jun 9;31(10):107739. (3) Aki S, Yoshioka K, Takuwa N, Takuwa Y. Mol Biol Cell. 2020 Mar 1;31(5):360-372.

anti-Involucrin (SY5, Invitrogen, USA) - Anti-Involucrin Monoclonal Antibody (SY5) (Product # MA5-11803) was validated in the cells HaCaT, A-431, HeLa, MDA-MB-231 and Raji by Western blot analysis and in Formalin-fixed, paraffin-embedded human skin by IHC. It was cited in 44 publications. Selected publications: (1) Cheung PY, Yip YL, Tsao SW, Ching YP, Cheung AL. J Cell Biochem. 2011 Jan;112(1):157-68. (2) Zhang Z, Zi Z, Lee EE, Zhao J, Contreras DC, South AP, Abel ED, Chong BF, Vandergriff T, Hosler GA, Scherer PE, Mettlen M, Rathmell JC, DeBerardinis RJ, Wang RC. Nat Med. 2018 May;24(5):617-627. (3) Uzgare AR, Isaacs JT. Cancer Res. 2004 Sep 1;64(17):6190-9.

anti-MTAP (4158S, Cell Signaling, USA) - MTAP Antibody #4158 was validated in the HT29 and NIH/3T3 cells by Western blot analysis. It was cited in 7 publications. Selected publications: (1) Du C, Hansen LJ, Singh SX, Wang F, Sun R, Moure CJ, Roso K, Greer PK, Yan H, He Y. Cell Rep. 2019 Sep 17;28(12):3199-3211.e5. (2) Gao G, Zhang L, Villarreal OD, He W, Su D, Bedford E, Moh P, Shen J, Shi X, Bedford MT, Xu H. Nucleic Acids Res. 2019 Jun 4;47(10):5038-5048. (3) Fedoriw A, Rajapurkar SR, O'Brien S, et al. Cancer Cell. 2019 Jul 8;36(1):100-114.e25.

anti-PRMT5 (2252S Cell Signaling, USA) - PRMT5/Skb1Hs Methyltransferase Antibody #2252 was validated in the HeLa, COS, PC12 and NIH3T3 cells by Western blot analysis. It was cited in 8 publications. Selected publications: (1) Braun CJ, Stanciu M, Boutz PL, et al. Cancer Cell. 2017 Oct 9;32(4):411-426.e11. (2) Clarke TL, Sanchez-Bailon MP, Chiang K, et al. Mol Cell. 2017 Mar 2;65 (5):900-916.e7. (3) Yang CY, Chiu LL, Chang CC, Chuang HC, Tan TH. FASEB J. 2018 Jun 19;32(12):fj201800244RR.

anti-MAT2A (Sc-166452, Santa Cruz, USA) - Anti-MAT Iα/IIα Antibody (B-10): sc-166452 was validated in the HeLa, PC-12, and NIH3T3 cells by Western blot analysis. It was cited in 2 publications. (1) . Wang, Z., et al. 2019. J. Sci. Food Agric. 99: 4849-4862. (2) Lambrecht, C., et al. 2020. Cancer Genomics Proteomics 17: 669-685.

anti-SDMA (13222S Cell Signaling, USA) - Symmetric Di-Methyl Arginine Motif [sdme-RG] MultiMab™ Rabbit mAb mix #13222 was validated in MCF7 cells, untreated (-) or treated with Adenosine-2', 3'-dialdehyde (AdOx, 100 µM, 24 hr; +). It was cited in 28 publications. Selected publications: (1) Liu F, Xu Y, Lu X, Hamard PJ, Karl DL, Man N, Mookhtiar AK, Martinez C, Lossos IS, Sun J, Nimer SD. Nucleic Acids Res. 2020 Apr 6;48(6):2956-2968. (2) Radzisheuskaya A, Shliaha PV, Grinev V, Lorenzini E, Kovalchuk S, Shlyueva D, Gorshkov V, Hendrickson RC, Jensen ON, Helin K. Nat Struct Mol Biol. 2019 Nov;26(11):999-1012. (3) Mersaoui SY, Yu Z, Coulombe Y, Karam M, Busatto FF, Masson JY, Richard S. EMBO J. 2019 Aug 1;38(15):e100986.

anti-Caspase-3 (5A1E, Cell Signaling, USA) - Cleaved Caspase-3 (Asp175) (5A1E) Rabbit mAb #9664 was validated in MCF7 cells C6 (rat), NIH/3T3 (mouse), and Jurkat (human) cells, untreated or treated with staurosporine #9953 (1uM, 3hrs) or etoposide #2200 (25uM, 5hrs) by Western blot analysis. It was cited in 2599 publications. Selected publications: (1) Zhang L, Jiang X, Pfau D, Ling Y, Nathan CF. J Exp Med. 2021 Feb 1;218(2):e20200887. (2) Xue Z, Lui VWY, Li Y, Jia L, You C, Li X, Piao W, Yuan H, Khong PL, Lo KW, Cheung LWT, Lee VHF, Lee AWM, Tsao SW, Tsang CM. J Exp Clin Cancer Res. 2020 Nov 26;39(1):262. (3) Beauchamp E, Yap MC, Iyer A, Perinpanayagam MA, Gamma JM, Vincent KM, Lakshmanan M, Raju A, Tergaonkar V, Tan SY, Lim ST, Dong WF, Postovit LM, Read KD, Gray DW, Wyatt PG, Mackey JR, Berthiaume LG. Nat Commun. 2020 Oct 22;11(1):5348.

anti-BAX (#2772, Cell Signaling, USA) - Bax Antibody #2772 was validated in HeLa cells and Bax knockout HeLa cells by Western blot analysis. It was cited in 983 publications. Selected publications: (1) Li Y, Zhang B, Xiang L, Xia S, Kucuk O, Deng X, Boise LH, Dong JT. Theranostics. 2020 Jun 18;10(17):7656-7670. (2) Ye K, Meng WX, Sun H, Wu B, Chen M, Pang YP, Gao J, Wang H, Wang J, Kaufmann SH, Dai H. Nat Commun. 2020 Jul 3;11(1):3301. (3) Upreti M, Galitovskaya EN, Chu R, Tackett AJ, Terrano DT, Granell S, Chambers TC. J Biol Chem. 2008 Dec 19;283(51):35517-25.

anti-p53 (Sc-126, Santa Cruz, USA) - Anti-p53 Antibody (DO-1): sc-126 was validated in A-431 and SW480 by Western blot analysis. It was cited in 5599 publications. Selected publications: (1) Schmoldt, A., et al. 1975. Digitoxin metabolism by rat liver microsomes. Biochem. Pharmacol. 24: 1639-1641. (2) Lee, J.W., et al. 2019. RUNX3 regulates cell cycle-dependent chromatin dynamics by functioning as a pioneer factor of the restriction-point. Nat. Commun. 10: 1897. (3) Liu, C., et al. 2019. Spindle assembly checkpoint inhibition can resensitize p53-null stem cells to cancer chemotherapy. Cancer Res. 79: 2392-2403. (4) Nassour, J., et al. 2019. Autophagic cell death restricts chromosomal instability during replicative crisis. Nature 565: 659-663.
anti-Actin (13E5, Cell Signaling, USA) - β-Actin (13E5) Rabbit mAb #4970 was validated in various cell lines including NIH3T3, HeLa, PAE and A431 by Western blot analysis. It was cited in 5599 publications. Selected publications: (1) Ramezani-Rad P, Chen C, Zhu Z, Rickert RC. Cell Rep. 2020 Nov 17;33(7):108403. (2) Wei D, Zhan W, Gao Y, Huang L, Gong R, Wang W, Zhang R, Wu Y, Gao S, Kang T. Cell Res. 2021 Feb;31(2):157-177. (3) Munkhbaatar E, Dietzen M, Agrawal D, et al. Nat Commun. 2020 Sep 10;11(1):4527.

anti-LMP1 mouse monoclonal antibody (CS.1-4, Dako, Agilent Technologies, USA) - Monoclonal Mouse Anti-Epstein-Barr Virus, LMP Clone CS.1-4 was validated in Burkitt lymphoma/leukemia and Hodgkin lymphoma cells by IHC using autostainer Link 48. References: (1) Pallesen G, Hamilton-Dutoit SJ, Rowe M, Young LS. Expression of Epstein-Barr virus latent gene products in tumour cells of Hodgkin's disease. Lancet 1991,337:320-2.(2) Vera-Sempere FJ, Burgos JS, Botella MS, Cordoba J, Gobernado M. Immunohistochemical expression of Epstein-Barr virus-encoded latent membrane protein (LMP-1) in paraffin sections of EBV-associated nasopharyngeal carcinoma in Spanish patients. Oral Oncol, Eur J Cancer 1996,32B:163-8. (3) Chung GT, Lou WP, Chow C, To KF, Choy KW, Leung AW, Tong CY, Yuen JW, Ko CW, Yip TT, Busson P, Lo KW. Constitutive activation of distinct NF-κB signals in EBV-associated nasopharyngeal carcinoma. J Pathol . 2013 Nov;231(3):311-22.

anti-PD-L1 antibody (clone 22C3, PD-L1 IHC 22C3; pharmDx assay; Agilent Technologies, USA). - PD-L1 IHC 22C3 pharmDx is a qualitative IHC assay intended for use in the detection of PD-L1 protein and was validated in the FFPE NSCLC, gastric or GEJ adenocarcinoma, ESCC, etc. Selected references: (1) Ma BBY, Lim WT, Goh BC, et al. Antitumor Activity of Nivolumab in Recurrent and Metastatic Nasopharyngeal Carcinoma: An International, Multicenter Study of the Mayo Clinic Phase 2 Consortium (NCI-9742). J Clin Oncol . 2018 May 10;36(14):1412-1418. (2) Keppens C, Dequeker EM, Pauwels P, Ryska A, 't Hart N, von der Thüsen JH. PD-L1 immunohistochemistry in non-small-cell lung cancer: unraveling differences in staining concordance and interpretation. Virchows Arch. 2020 Dec 4. doi: 10.1007/s00428-020-02976-5. Online ahead of print. (3) Naso JR, Wang G, Banyi N, Derakhshan F, Shokoohi A, Ho C, Zhou C, Ionescu DN. Comparability of laboratory-developed and commercial PD-L1 assays in non-small cell lung carcinoma. Ann Diagn Pathol. 2021 Feb;50:151590. (4) Krigsfeld GS, Prince EA, Pratt J, Chizhevsky V, William Ragheb J, Novotny J Jr, Huron D. Analysis of real-world PD-L1 IHC 28-8 and 22C3 pharmDx assay utilisation, turnaround times and analytical concordance across multiple tumour types. J Clin Pathol. 2020 Oct;73(10):656-664.

anti-HLA Class I A/B/C (ab7038, Abcam, USA) - Anti-HLA Class 1 ABC antibody [EMR8-5] (ab70328) was validated in the cell lines OSC20, HLA-A2402-transfected OSC20, K562 and recombinant HLA-A2402 heavy chain by western blot analysis and in a section of formalin-fixed paraffin-embedded normal human tonsil* by IHC which is performed on a Leica BONDTM system. It was cited in 93 publications. Selected publications: (1) Wojcik JB et al. Epigenomic Reordering Induced by Polycomb Loss Drives Oncogenesis but Leads to Therapeutic Vulnerabilities in Malignant Peripheral Nerve Sheath Tumors. Cancer Res 79:3205-3219 (2019). (2) Wei J et al. Ribosomal Proteins Regulate MHC Class I Peptide Generation for Immunosurveillance. Mol Cell 73:1162-1173.e5 (2019). (3) Ono T et al. Changes in immune parameters between pre-treatment and recurrence after (chemo) radiation therapy in patients with head and neck cancer. Sci Rep 10:11973 (2020).

anti-MHC Class-II (ab55152, Abcam, USA) - Anti-MHC Class II antibody [6C6] (ab55152) was validated in paraffin embedded human lymph node and bowel tissue sections by Immunohistochemistry and in the MHC Class II transfected 293T cells and non-transfected 293T cells by Western blot analysis. It was cited in 14 publications. Selected publications: (1) Damala M et al. Encapsulation of human

limbus-derived stromal/mesenchymal stem cells for biological preservation and transportation in extreme Indian conditions for clinical use. Sci Rep 9:16950 (2019). (2) Dufour A et al. C-terminal truncation of IFN-? inhibits proinflammatory macrophage responses and is deficient in autoimmune disease. Nat Commun 9:2416 (2018). (3) Kakavand H et al. PD-L1 Expression and Immune Escape in Melanoma Resistance to MAPK Inhibitors. Clin Cancer Res 23:6054-6061 (2017).

Eukaryotic cell lines

Policy information about cell lines

Cell line source(s)	The NPC and immortalized NP cell lines and NPC patient derived xenografts used in the study were established by the authors.
Authentication	All of the cell lines and patient derived xenografts have been authenticated by STR profiling.
Mycoplasma contamination	All the cell lines have been tested and confirmed to be negative for mycoplasma contamination.
Commonly misidentified lines (See ICLAC register)	None.

Animals and other organisms

Policy information about studies involving animals; ARRIVE guidelines recommended for reporting animal research

Laboratory animals	3- to 4-week-old female athymic nude mice with a stating weight of around 18-22 g were used in this study. Mice were kept within a specific animal room limits of 20 - 23 degree C and 40-60% humidity. The room run on a 12 hour light/dark cycle that from 7 am to 7 pm.
Wild animals	No wild animals were used in the study.
Field-collected samples	no field collected samples were used in the study.
Ethics oversight	All animal care and experimental procedures were approved by the University Animal Experimentation Ethics Committee (AEEC), the Chinese University of Hong Kong. The animal license was obtained from the Hong Kong Government, Department of Health.

Note that full information on the approval of the study protocol must also be provided in the manuscript.

Human research participants

Policy information about studies involving human research participants

Population characteristics	All patients included were diagnosed with nasopharyngeal carcinoma and treated using standard of care at the Prince of Wales Hospital, The Chinese University of Hong Kong, Hong Kong SAR, China. The cohort included 45 males, 18 females, median age of 54 years old. Additional clinical characteristics are included in the supplemental data of the current report.
Recruitment	The patients were randomly recruited from the Department of Clinical Oncology and Otorhinolaryngology, Head and Neck Surgery, Prince of Wales Hospital, The Chinese University of Hong Kong without any biases and other impact results.
Ethics oversight	The study protocol was approved by the Joint Chinese University of Hong Kong-New Territories East Cluster Clinical Research Ethics Committee, Hong Kong.

Note that full information on the approval of the study protocol must also be provided in the manuscript.

Flow Cytometry

Plots

Confirm that:

- [x] The axis labels state the marker and fluorochrome used (e.g. CD4-FITC).
- [x] The axis scales are clearly visible. Include numbers along axes only for bottom left plot of group (a 'group' is an analysis of identical markers).
- [x] All plots are contour plots with outliers or pseudocolor plots.
- [x] A numerical value for number of cells or percentage (with statistics) is provided.

Methodology

Sample preparation	Immortalized NPE cells were infected with a green fluorescent protein (GFP)-tagged recombinant EBV (Akata strain). After co-culturing of Akata cells and immortalized NPE cells for 48 hours, the infected epithelial cells were trypsinized, and then neutralized with medium containing 10% FBS. The resultant cell suspension was then centrifuged at 1500 rpm, 4خ for 5 min before discarding the supernatant and re-suspending the cell pellets in 1×PBS containing 2% FBS. The re-suspending cells

	were passed through a 100µm cell strainer and subjected to fluorescent-activated cell sorting (FACS) using a BD FACSARIA III system (BD biosciences, USA) for isolating GFP(EBV)-positive cells. The isolated EBV-positive cells were then cultured for 7 or 14 days. Growth media were replaced every 48 hours. At respective time points, cells were harvested and the percentage of EBV-positive cells was determined using the BD FACSARIA III system.
Instrument	Both FACS sorting and FACS analysis were performed with BD FACSARIA III system (excitation: 488 nm; emission: 530/30nm)
Software	Data was acquired and analysed using BD FACSDiva software v8.0.2
Cell population abundance	Sorted populations were >90% pure which is determined by the positivity of the GFP signal in the sorted cells.
Gating strategy	For detecting and isolating the EBV-positive cells, the gating strategy is P1: SSC-A vs FSC-A (for identifying cell population), P2: FSC-W vs FSC-H (for identifying single cell population), P3: SSC-W vs SSC-H (for identifying cell population), and P4: SSC-A vs GFP-A (for gating cells with high GFP signals). (Supplementary Figure S8C)

☒ Tick this box to confirm that a figure exemplifying the gating strategy is provided in the Supplementary Information.
